# High-performance magnetic artificial silk fibers produced by a scalable and eco-friendly production method

**DOI:** 10.1007/s42114-024-00962-y

**Published:** 2024-10-02

**Authors:** Gabriele Greco, Benjamin Schmuck, Lucia Del Bianco, Federico Spizzo, Luca Fambri, Nicola Maria Pugno, Sabino Veintemillas-Verdaguer, Maria Puerto Morales, Anna Rising

**Affiliations:** 1 Department of Animal Biosciences, Swedish University of Agricultural Sciences, Box 7011, 75007 Uppsala, Sweden; 2 Department of Medicine Huddinge, Karolinska Institutet, Neo, 14183 Huddinge, Sweden; 3 Department of Physics and Earth Science, University of Ferrara, Via G. Saragat 1, 44122 Ferrara, Italy; 4 Department of Industrial Engineering and INSTM Research Unit, University of Trento, via Sommarive 9, 38123 Trento, Italy; 5 Department of Civil, Environmental and Mechanical Engineering, Laboratory for Bioinspired, Bionic, Nano, Meta Materials & Mechanics, University of Trento, Via Mesiano 77, 38123 Trento, Italy; 6 School of Engineering and Materials Science, Queen Mary University of London, UK, Mile End Road, London, E1 4NS UK; 7 Instituto de Ciencia de Materiales de Madrid, ICMM/CSIC, Sor Juana Inés de la Cruz 3, 28049 Madrid, Spain

**Keywords:** Bio-fibers, Mechanical properties, Wet-spinning, Composite, Nanomaterials, Superparamagnetism

## Abstract

Flexible magnetic materials have great potential for biomedical and soft robotics applications, but they need to be mechanically robust. An extraordinary material from a mechanical point of view is spider silk. Recently, methods for producing artificial spider silk fibers in a scalable and all-aqueous-based process have been developed. If endowed with magnetic properties, such biomimetic artificial spider silk fibers would be excellent candidates for making magnetic actuators. In this study, we introduce magnetic artificial spider silk fibers, comprising magnetite nanoparticles coated with meso-2,3-dimercaptosuccinic acid. The composite fibers can be produced in large quantities, employing an environmentally friendly wet-spinning process. The nanoparticles were found to be uniformly dispersed in the protein matrix even at high concentrations (up to 20% w/w magnetite), and the fibers were superparamagnetic at room temperature. This enabled external magnetic field control of fiber movement, rendering the material suitable for actuation applications. Notably, the fibers exhibited superior mechanical properties and actuation stresses compared to conventional fiber-based magnetic actuators. Moreover, the fibers developed herein could be used to create macroscopic systems with self-recovery shapes, underscoring their potential in soft robotics applications.

**Supplementary information:**

The online version contains supplementary material available at 10.1007/s42114-024-00962-y.

## Introduction

Actuators find applications in soft robotics, medical devices, and wearables. However, there is a large need for innovative, environmentally friendly materials capable of being crafted into devices with milli/micrometric dimensions [1, 2]. These materials must possess dual characteristics, inducing strain and exhibiting mechanical resilience while retaining strength throughout their operation [3–5]. Fibrous magnetic materials are particularly intriguing since they can generate mechanical motion without being tethered to an external power source [6–9].

Typically, synthetic polymers are used for making actuators, offering softness but often lacking high strength and Young’s modulus [6, 10–12]. In this context, native silk presents a promising solution as it combines softness and desirable mechanical properties [13, 14], and indeed, native silk has shown great potential as magnetic actuator materials [15–17]. However, challenges arise from the difficulty in producing native silk materials in large quantities, attributed to the cannibalistic nature of spiders and the negative environmental impact of silkworm cultivation [18, 19]. Despite these challenges, native silk–nanomaterial composites have been designed but the need for post-spinning treatment resulted in several setbacks, e.g., excessive fiber plasticization caused by the solvent in which the nanomaterials are dispersed [14, 16, 20, 21].

An alternative to the use of native silk would be to turn to recombinant production methods to produce the silk proteins and artificially spin the silk fibers. This alternative comes with concerns related to production levels and costs [22], but recent technological advancements have showcased the scalability of producing a small genetically engineered spider silk protein (NT2RepCT) [23], so-called minispidroin, in bioreactor cultivations [24]. This minispidroin can also be purified and spun into artificial silk fibers using an eco-friendly protocol [24]. Continuous fibers made from this minispidroin have micrometric diameters, are cytocompatible [25], and they possess good mechanical properties that can be tuned by altering the spinning conditions [26, 27]. However, they lack magnetic properties, posing a challenge for actuator applications.

An interesting way forward would be to design a composite made from recombinant minispidroins and magnetic nanomaterials [28]. Preferably, the nanomaterials would be mixed with the spinning dope (soluble minispidroins) before spinning to enable the generation of composite fibers with homogeneously distributed nanomaterials, which is necessary to avoid reducing fiber’s mechanical properties [29, 30]. Early attempts to spin artificial silk fibers containing nanomaterials have only resulted in fibers with compromised mechanical properties, possibly due to that the methods employed rely on harsh solvents that are incompatible with natively folded proteins [31–35]. The use of water as the sole solvent to make protein-nanomaterial composites is challenging because the nanomaterials are not easily dispersed in a range of pH 6–8. However, magnetite nanoparticles coated with meso-2,3-dimercaptosuccinic acid (DMSA) are dispersed well in aqueous solutions, also at physiologically relevant pHs [36].

Herein, we pioneer the design and manufacturing of magnetic artificial spider silk fibers using only water-based solutions. The composite fibers were easily spun at a scale of hundreds of meters and maintained their mechanical properties even at high magnetite concentrations (up to 20% w/w). Notably, they offer tunable magnetic functionality and exhibit the highest actuation stress among described magnetic fiber actuators. Their versatility extends to forming self-supporting macro devices, enhancing their potential applications.

## Results and discussion

**Fig. 1:**
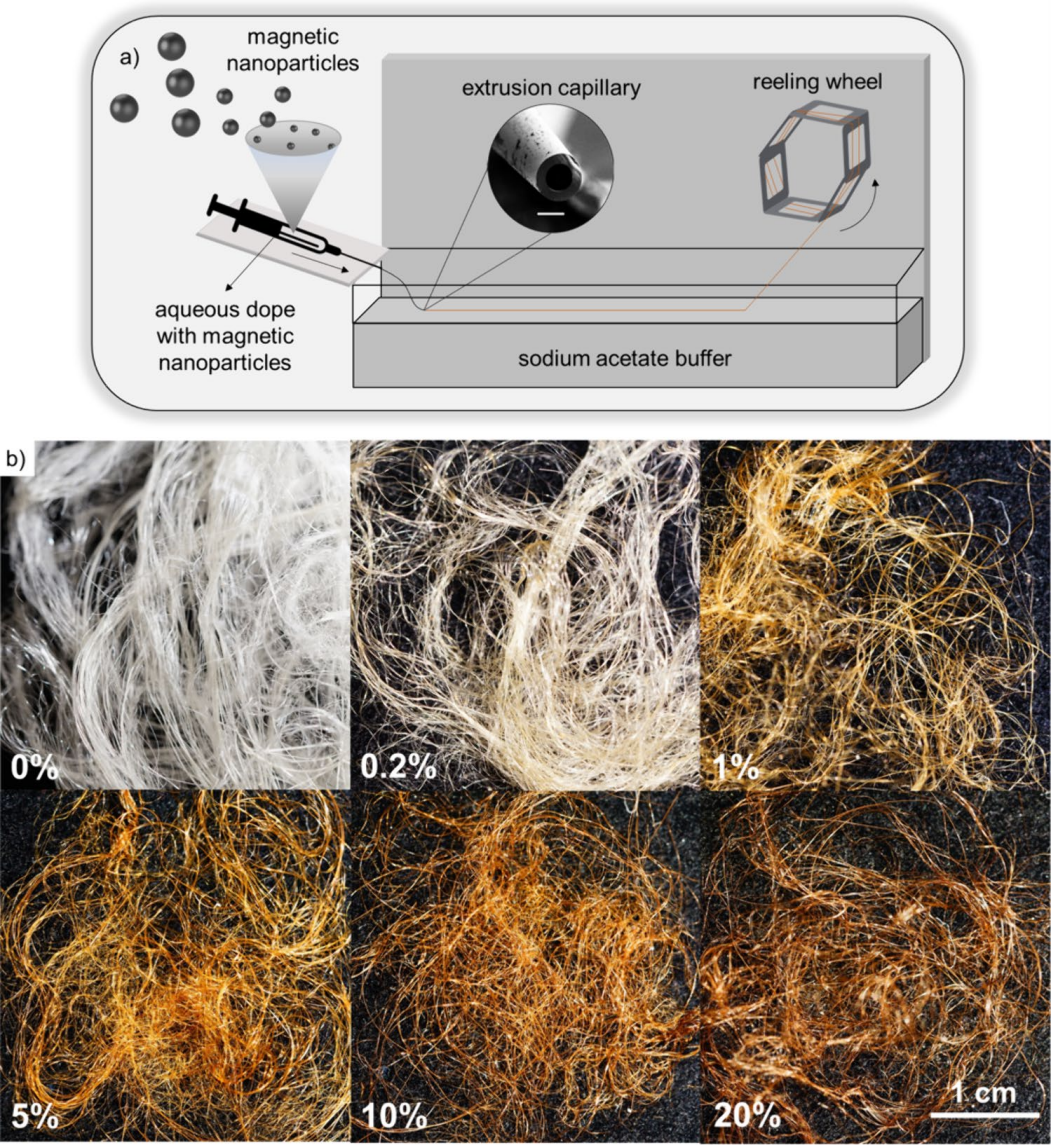
Spinning of the magnetic artificial silk fibers. **a** Schematic of the spinning setup. The nanoparticles were mixed with the recombinant spider silk protein solution (300 mg/mL in Tris–HCl pH 8) to generate spinning dopes containing 0.2–20% w/w magnetite. The solutions were spun into fibers by extrusion through a glass capillary into a 0.75 M NaAc buffer at pH 5 [27]. The fibers were reeled at constant speed by a rotating wheel. The spinning was continuous and allowed the collection of bundles of magnetic silk fibers. The scale bar for the capillary in **a** is 20 µm. **b** Pictures of composite fibers containing different nominal w/w magnetite concentrations

To achieve a uniform dispersion of nanomaterials in the protein spinning dope, we used magnetite nanoparticles that were coated with meso-2,3-dimercaptosuccinic acid (DMSA), which makes the surface negatively charged and hydrophilic [36]. The nanoparticles had a mean diameter of ~ 18 nm (Fig. S1, Table S1) and could successfully be dispersed in aqueous solutions. Composite artificial spider silk fibers were produced using the recombinant spider silk protein NT2RepCT [23], which can be produced in scalable bioreactor cultivations at significant yields ($\sim$ 15 g of pure protein per liter of microbial cell culture) and spun into artificial silk fibers with an eco-friendly protocol using water as the sole solvent [24]. Composite spinning dopes containing 0.2–20% w/w magnetite were extruded with a syringe pump via a pulled glass capillary into a bath containing an acetate buffer at pH 5 (Fig. 1a). The spinning of the protein-nanoparticle solution was continuous, which made it possible to produce hundreds of meters of fibers and collect them into bundles. Notably, the spinning process remained effective even at a magnetite concentration of 20% w/w (Fig. 1b, Supplementary video 1). This is impressive considering that previously reported manufacturing methods have resulted in silk composites with less than 2% w/w nanoparticles (Table S2). High concentrations of nanoparticles in fibrous composites can also be achieved through electrospinning [37–40]. However, these methods typically use organic solvents, such as chloroform, which further underscores the quality of our approach.

**Fig. 2:**
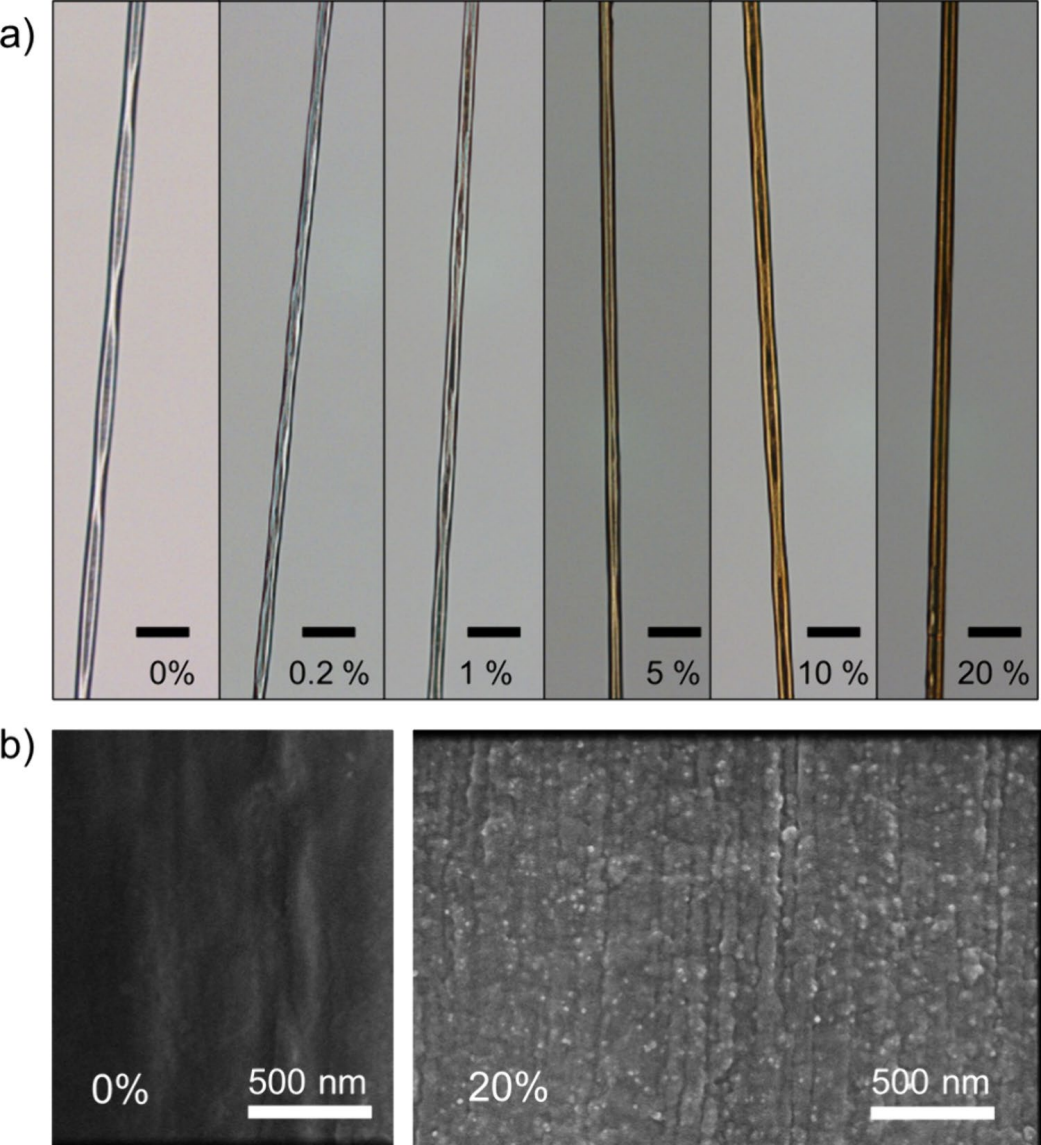
**a** Representative light microscopy images of the magnetic artificial silk fibers with different nominal w/w magnetite concentrations. Scale bars are 50 µm. **b** High magnification SEM images of the NT2RepCT fibers (i.e., fibers containing no nanoparticles) and 20% w/w (nominal) of magnetite, which indicates homogeneous dispersion of the nanoparticles

The presence of nanoparticles did not affect the overall morphology of the fibers (Fig. 2a and Fig. S2), which displayed an average diameter between 8 and 15 µm. High magnification and resolution SEM images indicated that the magnetite nanoparticles were homogeneously dispersed in the fibers (Fig. 2b and Fig. S3). Furthermore, results from energy-dispersive X-ray (EDX) analysis confirmed that iron was found in the protein matrix in a stoichiometric ratio compatible with the nominal values, even at the highest concentration (Fig. S4). This is noteworthy considering the natural tendency of nanoparticles to form agglomerates [41] and prompted us to obtain further evidence using magnetic measurements.

**Fig. 3:**
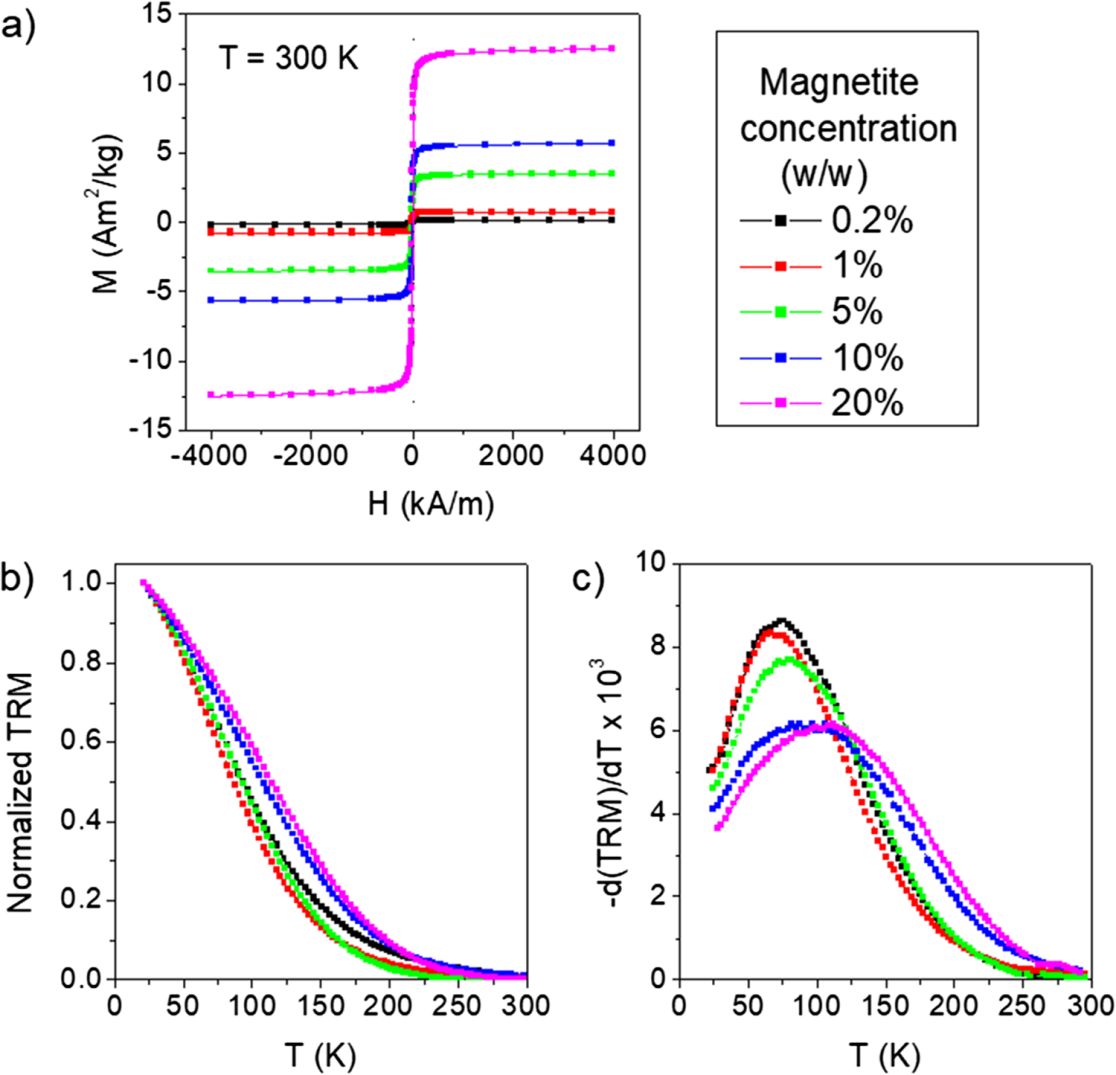
Magnetic properties of the magnetic artificial silk fibers. **a** Magnetization *M* vs. magnetic field *H* measured at *T* = 300 K (curves corrected for the magnetic signal from the NT2RepCT matrix; see “Materials and methods” and Fig. S15). **b** Thermoremanent magnetization (TRM) vs. *T* (magnetic field previously applied to the samples *H* $=$ 4 kA/m); the curves are normalized to their initial value at *T* = 20 K. **c** Derivatives of the curves in **b**, normalized to their area. The different colors indicate different nominal w/w magnetite concentrations

The magnetic properties of the composite fibers were studied by SQUID magnetometry methods, which also informed on inter-particle interaction effects (Figs. 3 and S5–S7, supplementary section S1). Magnetic loops were measured at different temperatures (Figs. 3a and S6), and, by comparing the value of the saturation magnetization to that of the DMSA-coated nanoparticles alone (Fig. S5), we estimated the concentration of magnetite in the fibers. These estimates were in good agreement with the nominal values for all samples (Table S3). At 300 K, no magnetic hysteresis was observed in the composite fibers, i.e., coercivity and remanent magnetization were null (Fig. 3a), in contrast to what was found at 5 K (Fig. S6). This result is compatible with a superparamagnetic behavior of the nanoparticles at room temperature. The magnetic moment of an isolated single-domain nanoparticle can undergo magnetic relaxation, possibly culminating in the superparamagnetic behavior at a critical temperature (blocking temperature) at which the thermal energy is comparable to the magnetic energy required for its reversal [42]. The onset of the superparamagnetic regime implies that the nanoparticle magnetic moment flips randomly between different spatial orientations.

Next, the magnetic relaxation of the nanoparticles in the fibers was studied through the analysis of the thermoremanent magnetization vs. temperature and corresponding temperature derivative curve [42]. The latter is a figure of the distribution of blocking temperature values for the assembly of embedded nanoparticles. In all samples, the thermoremanent magnetization went to zero with increasing temperature (Fig. 3b), confirming the progressive entrance into the superparamagnetic regime of the whole nanoparticle assembly. For magnetite concentrations up to 5% w/w, the derivative curves in Fig. 3c feature a narrow profile, and the peak temperature coincides with the blocking temperature predicted for the magnetite nanoparticles when considered individually relaxing magnetic elements (more details can be found in section S1). This indicates that the nanoparticles are homogeneously dispersed in the protein matrix and sufficiently distant from each other to make dipolar interactions negligible. The derivative curves of fibers containing 10% and 20% w/w magnetite were broader and slightly shifted to higher temperatures, which is consistent with the presence of inter-particle dipolar interactions and suggests that the nanoparticles are well distributed spatially although not as homogeneously as in fibers with lower magnetite concentration [42, 43]. This was further supported by the results of the field dependence of the remanent magnetization (Δ*M* plots analysis at 20 K, Fig. S7). Therefore, despite inter-particle dipolar interactions, which in the samples with higher magnetite concentration slightly increase the blocking temperature, the whole nanoparticle assembly in all fibers is in the superparamagnetic regime at room temperature. This implies that the composite fibers are not permanently magnetized, but their magnetic response can be switched on and off by an external magnetic field. This could be an important property for materials intended for soft robotics applications for which the material must also have sufficient mechanical properties to withstand the stresses associated with completion of the intended task [7, 8]. For this reason, we investigated the mechanical properties of the composite fibers.

**Fig. 4:**
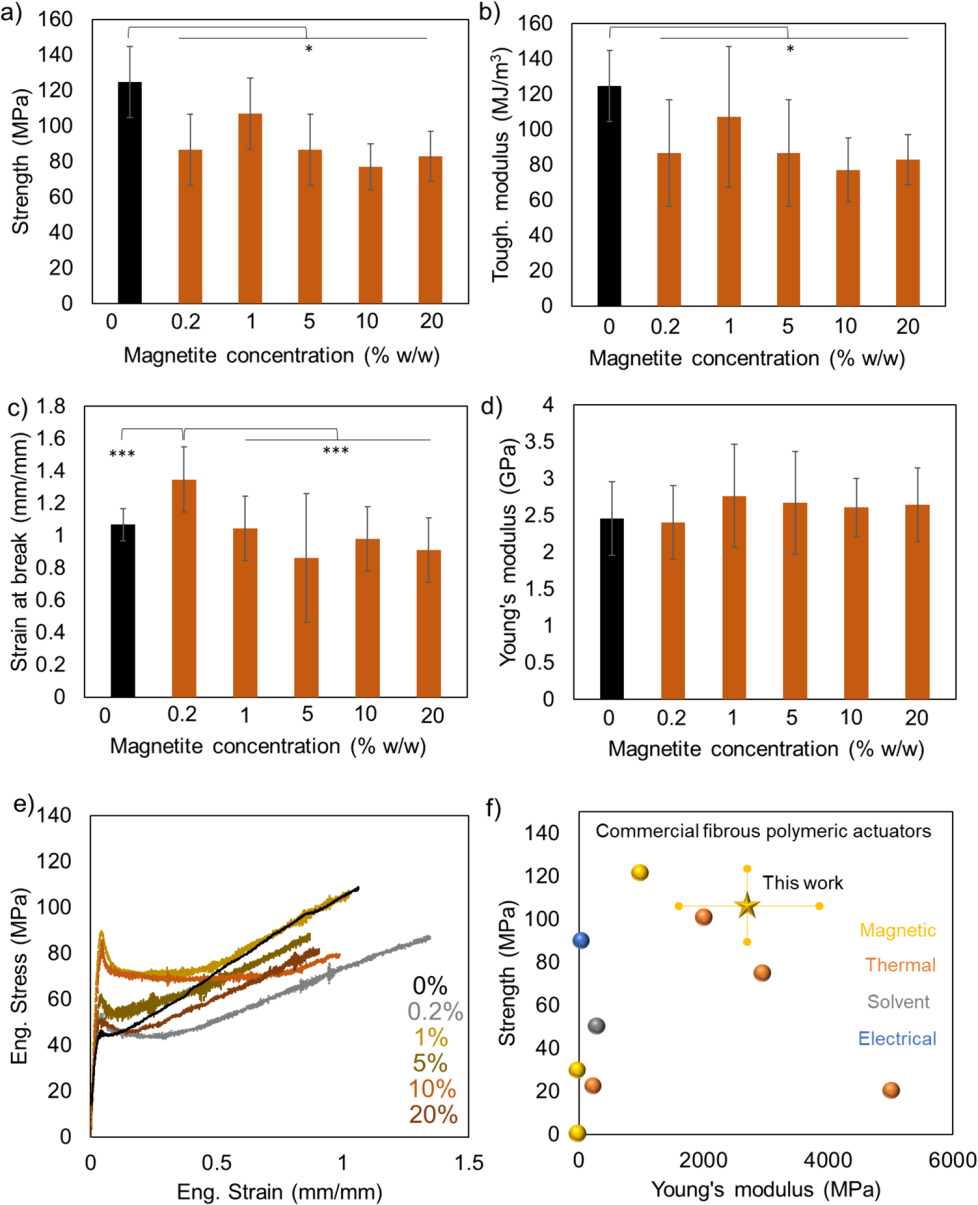
Mechanical properties of the magnetic artificial silk fibers. **a** Strength, **b** toughness modulus, **c** strain at break, **d** Young’s modulus. The values of the mechanical properties are here reported vs. nominal values of magnetite concentrations. Asterisk (*) indicates *p* < 0.05, **p < 0.01, and ***p < 0.001. **e** Representative stress-strain curves of the NT2RepCT and composite fibers that were tensile tested. **f** Ashby plot of the mechanical properties of commercial polymeric fiber actuators and the mechanical properties of the actuator developed in this work. The data from the literature are from references [9, 44–51]. The actuation of the strain was induced by electrical (blue), thermal (orange), and magnetic stimuli (yellow) or when the fibers were immersed in a solvent (gray)

Nanomaterial composites often display reduced strength, maintained Young’s modulus, and a strain at break that can be higher or lower compared to the pristine material [32, 52–59]. This is due to that the nanomaterials act as defects in the composite when there is no or poor mechanical communication between the nanomaterials and the surrounding matrix [60]. In line with this, the strength and the toughness modulus of the composite fibers were reduced compared to fibers without nanoparticles (Fig. 4a, b). Surprisingly, at magnetite concentrations higher than 0.2% w/w, no further deterioration of the strength and toughness modulus was detected (Figs. 4, S8–S9, and datasheet). The highest strain at break for all fibers investigated was obtained for fibers containing 0.2% w/w magnetite which remains to be rationalized but agrees with previous findings for silk composites (Fig. 4c, Supplementary Table S2). Young’s modulus of the composite fibers was not significantly affected compared to that of the pristine material (Fig. 4d).

In order to better understand the effects of the nanoparticles on the mechanical properties of the composite, we analyzed our results using the model developed by Zare [29] for Young’s modulus and strength of the composite fibers (see supplementary section S2, Figs. S10–S11). In this model, specific attention is devoted to the role of the interphase between the matrix (in our case NT2RepCT) and the filler (magnetite core of the nanoparticles). The interphase is in this case represented by the interface between the magnetite and the DMSA, the DMSA coating, and the interface between the DMSA and the silk proteins. According to the model, the mechanical strength and stiffness of the interphase (*σ*_*interphase*_ and *E*_*interphase*_, respectively) as well as its thickness (*t*, which is estimated to be less than 1 nm for our nanoparticles) influence the resulting mechanical properties of the composite material (Fig. S10a). If the strength and the stiffness of the interphase are much lower than those of the matrix, the presence of the nanoparticles (up to 20% w/w magnetite concentration) will reduce the strength of the composite while leaving Young’s modulus unaffected, which agrees with the experimental data (Fig. S10b-e, see supplementary section S2 for details regarding the choice of values for *E*_*interphase*_, *σ*_*interphase*_, and *t*). For Young’s modulus, the model shows that the values of *E*_*interphase*_ must be much higher than those we used to fit the model to significantly stiffen the composite fibers with respect to the pristine material (Fig. S10b). Likewise, the concentration of magnetite has to be much higher than 20% w/w to induce a further decline of the strength of the composite fiber with respect to the pristine material, which aligns well with the experimental data (Fig. S10c–e). Thus, the experimental values we obtained for the mechanical properties of the composite fibers can be explained by the model when using low values for the interphase mechanical properties and implies that there is only a weak mechanical coupling between the nanoparticles and the surrounding protein matrix.

Another way of investigating the level of mechanical coupling between the nanoparticles and the protein matrix could be to analyze the shape of the stress-strain curves and, in particular, the presence and degree of necking. For polymeric fibers, necking is associated with the presence of defects in the material since these alter the distribution of the stress, leading to localized deformations and thus an overall reduction of the measured load [61, 62]. Indeed, the fibers containing the nanoparticles displayed a higher degree of necking compared to the control, supporting the idea that there is only a weak mechanical coupling between the protein matrix and the magnetite, thus reducing the strength of the composite (Fig. S12). The lack of mechanical coupling could be due to the absence of interaction between the protein matrix and the nanoparticles, which is also supported by the fact that the protein solution remained soluble after the addition of the nanoparticles. In the case of strong interactions between the two, the nanoparticles would have acted as cross-linkers, likely causing aggregation of the proteins or solidification of the spinning dope. The absence of any significant interaction between the DMSA-coated nanoparticles and the proteins was further confirmed using a surface plasmon resonance assay (see supplementary section S3 and Fig. S13). The lack of interaction also supports the notion that the nanoparticles do not alter the secondary structure of the silk in the composite fiber. For instance, a recent study on silk fibroin film mixed with iron oxide nanoparticles, using Fourier-transform infrared spectroscopy analysis, confirmed that the nanoparticles do not affect the secondary structure of the silk [63].

While the weak interactions between the nanoparticles and the protein matrix in the fibers can explain the reduced strength of the composite fibers compared to the pristine fibers, the composite fibers presented herein have outstanding mechanical properties compared to commercial fibers used for actuation in soft robotics, especially when considering magnetic actuators (Fig. 4e, f) [6–9, 44–51].

**Fig. 5:**
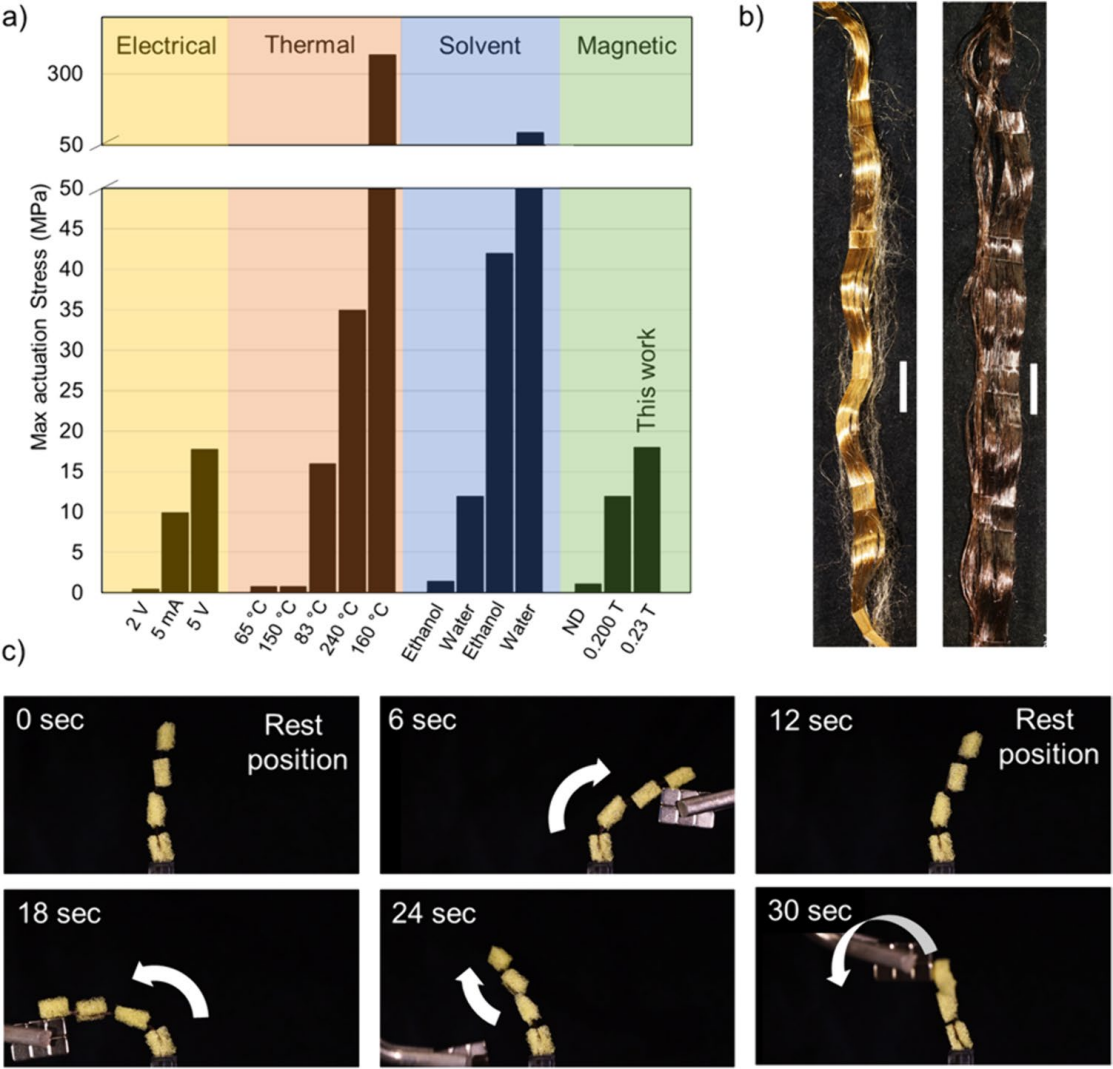
Actuation properties of the magnetic artificial silk fibers. **a** Maximum actuation stresses measured for fibrous actuators induced by electrical, thermal, and magnetic stimuli or when the fibers were immersed in a solvent. ND means that the actuating magnetic field was not declared. The material presented in this work has the highest actuation stress among magnetic fibrous actuators. The data were obtained from the following references [15, 44–51, 64–68]. **b** Bundles of magnetic artificial silk fibers. Scale bars are 2 cm. **c** Proof of concept that the magnetic fibers can be used for the design of a macroscopic actuator device. In this case, a bundle of magnetic silk fibers (10% w/w magnetite concentration) was activated by a magnet which induced a finger-like motion. When the magnet was removed, the fiber bundles retained their original shape

The properties of the composite fibers presented here make them interesting for applications in soft robotics. As a proof of their usefulness, we performed magnetic actuation experiments on single fibers, using a custom-made setup presented previously [16]. From these experiments, the actuation stress generated in the composite fibers when exposed to an external magnetic field gradient was determined. In general, actuation stresses in fibers can be obtained by immersion in a solvent [15, 45, 67, 68], application of a voltage [44, 64, 65], or exposure to high temperatures [48–51, 66] or magnetic fields (Fig. 5a). When comparing the performance of our magnetic spider silk fibers to the fibrous magnetic actuators described in literature for which the actuation stress was measured, it is evident that the actuation stress mediated by our fibers is impressive (Fig. 5a) [16, 46, 47, 69]. This combined with the fibers’ good mechanical properties (Fig. 4) also in relation to other materials used for actuation (Fig. 4e, f and Fig. S14) [15, 44–51, 64–69] encouraged us to build a device from a bundle of fibers and sponge cubes, which mimics a robotic finger (Fig. 5b, c). The device was self-supporting and responded to magnetic actuation (Fig. 5c and Supplementary video 2). Importantly, the induced actuation was reversible, and the system displayed a spontaneous shape recovery which is a desirable characteristic for specific actuators. This creates a solid foundation for developing future in-depth studies focused on the magnetic actuation properties of our fibers [70].

To summarize, the negative environmental impact of materials used in soft electronics and robotics has to be tackled at the design level [4, 5, 71]. In line with this, we herein present a manufacturing process of spider silk composite fibers that employ only water-based solvents and ambient temperature. The composite fibers have properties that outperform most currently used fibrous magnetic actuators and can be produced in large quantities. Given the tunable material properties, fiber dimensions, and actuation capabilities, magnetic artificial spider silk fibers should serve well for several applications in the soft robotics and related fields.

## Concluding remarks

In this work, we develop a novel fiber—a magnetic artificial spider silk fiber—designed with a specific focus on soft robotics applications. These fibers exhibit high strength and flexibility, offering a sustainable, large-scale production process that avoids the need for harsh chemicals. Notably, their magnetic characteristics and exceptional actuating stress levels surpass the performance of conventional fibrous magnetic actuators. Consequently, these fibers possess the essential attributes necessary for a wide range of applications, particularly in scenarios demanding untethered device operation.

## Materials and methods

### Synthesis and coating of the nanoparticles

Nanoparticles were produced and coated as described in Ovejero et al. [72] and Roca et al. [73], respectively. Briefly, magnetite nanoparticles were synthesized by thermal decomposition of Fe(acac)_3_ in the presence of oleic acid and oleylamine as surfactants and organic solvents with different boiling points. In particular, iron(III) acetylacetonate 99% (Acros Organics, Geel, Belgium) was decomposed in benzyl ether (99%; Acros Organics, Geel, Belgium) in the presence of oleic acid (OA; 80%; GPR Rectapur®, VWR, Leicestershire) and 1,2-dodecanediol 90% (ODA; Sigma-Aldrich, San Luis, MO, USA). We used a molar ratio of 1:3:2 (Fe(acac)_3_:OA:ODA) and a concentration of the iron precursor of 0.1 M. The solution was heated up to 200 °C for 120 min with mechanical stirring and under a nitrogen flow. Then, we heated the solution to reflux (bp 254 °C) for 30 min in an N_2_ atmosphere. Once at room temperature, the solution was mixed with ethanol and centrifuged at 5650 g for 10 min. The supernatant was then discarded. Lastly, we mixed the nanoparticles with 40 mL of hexane and 0.1 mL of oleic acid and centrifuged twice at 5650* g* for 10 min. After this, we obtained a stable hydrophobic suspension. Then, we employed a ligand exchange reaction of oleic acid for dimercaptosuccinic acid (DMSA) to make magnetite nanoparticles hydrophilic and thus dispersible in water. We first coagulated the nanoparticles from the hydrophobic suspension by adding ethanol and centrifugation at 2825* g* for 10 min to eliminate the solution. Then, we added a solution of toluene (25 mL) and a solution of 90 mg DMSA in 5 mL of dimethyl sulfoxide (DMSO) sonicated for 5 min and mechanically stirred for 24 h. Then, we added toluene and centrifuged again, discarding the supernatant containing the oleic acid–coated particles. We then mixed and centrifuged with ethanol and acetone several times to remove the free oleic acid molecules. The nanoparticles were then dispersed in alkaline water and then dispersed at pH 7. The dispersion was then dialyzed and filtered through a 0.2 µm pore size syringe.

The magnetite nanoparticles were characterized by transmission electron microscopy (TEM, JEOL JEM 1011, Peabody, MA, USA), X-ray powder diffraction (XRD, Bruker D8 Advance, Billerica, MA, USA), thermogravimetric analysis (TG-DTA, Q600 TA Instruments, New Castle, DE 19720, USA), and photon correlation spectroscopy (PCS, Zetasizer Nano, Malvern Panalytical, Malvern, UK) (Fig. S1, Table S1).

### Spinning the artificial silk (w and w/o nanoparticles)

The minispidroin NT2RepCT [23] (with 6xHis-tag) was expressed in a bioreactor in a fed-batch culture and purified in native conditions with a 20 mM Tris–HCl buffer at pH 8 and by using immobilized ion metal chromatography (IMAC) as described earlier [24]. After elution of NT2RepCT from the IMAC column with 20 mM Tris–HCl and 200 mM imidazole, NT2RepCT was dialyzed against 20 mM Tris–HCl (pH 8). To prepare the spinning dope, NT2RepCT was concentrated to ~ 300 mg/mL (which represents an optimum in order to maximize the mechanical properties of the fiber [27]) with an Amicon Ultra-15 centrifugal filter unit (Merck-Millipore, Darmstadt, Germany) at 4000 × g and 4 °C using an ultracel-10 membrane (10 kDa cutoff). After recovering the spinning dope from the centrifugal filter unit and transferring 500 µL to a new filter unit, DMSA-coated magnetite nanoparticles in an H_2_O suspension (12–50 mg/mL Fe_3_O_4_) were added. The NT2RepCT and magnetite blend were re-concentrated to a final volume of 500 µL, corresponding to the original volume of the spinning dope before adding the magnetite suspension to the centrifugal filter unit. Although it was difficult to judge due to the very viscous consistency and blackness of the spinning dope, independent of the nanoparticle concentration, no agglomerates were observed with the naked eye. Thus, the dope was prepared in a way to expect a final protein concentration of 300 mg/mL in the spinning dope, while expecting a magnetite (Fe_3_O_4_) concentration in the dry fiber of 0.2%, 1%, 5%, 10%, or 20% w/w. Then, the final dope was transferred into a 1-mL syringe with a Luer lock tip (BD, Franklin Lakes, NJ, USA). To make fibers, a recently developed optimized spinning protocol was used [27]. The syringe with the spinning dope was mounted in a neMESYS low-pressure (290 N) syringe pump (CETONI, Korbußen, Germany) where it was connected to a pulled glass capillary via polyethylene tubing. The glass capillary had a tapered tip with an orifice diameter of 50 ± 10 µm and was used to extrude the NT2RepCT/magnetite blend into a spinning bath containing 4 L 0.75 M acetate (Na) at pH 5. As soon as the spinning dope entered the spinning bath, it solidified, and a fiber was formed. The fibers were continuously collected at the end of the 0.8 m long bath with a rotating wheel at 0.58 m/s.

### SEM and EDX

A Zeiss SUPRA 40 field emission scanning electron microscope was used to investigate the morphology of the fibers with the secondary electron detectors. The samples were coated with an alloy Pt:Pd (80:20) utilizing a Quora Q150 and mounted on a standard Zeiss stab. Further, SEM and EDX characterization was performed with a FEI VERIOS 460 and an EDAX Octane Plus.

### Magnetic measurement

The magnetic study was carried out using a Quantum Design MPMS-XL superconducting quantum interference device (SQUID) magnetometer, which can measure the magnetic moment of a sample as a function of magnetic field (maximum applied field *H* = 4 × 10^3^ kA/m) and temperature *T* (5–300 K range). To calculate the magnetization *M* (magnetic moment/sample mass), the mass of the sample was measured with a precision of 10^−8^ kg. The fibers loaded with DMSA-coated nanoparticles were measured by taking a certain amount of material and manually forming a small bundle, which was inserted directly into the sample holder for SQUID supplied by Quantum Design. NT2RepCt fibers without magnetic nanoparticles were also analyzed as reference material; DMSA-coated nanoparticles alone were measured in powder form.

The spidroin matrix exhibited a paramagnetic behavior at very low temperatures, while at *T* = 300 K a diamagnetic signal predominated (Fig. S15). Therefore, to estimate the weight fractions of magnetite in the fibers, first, the hysteresis loops of the samples—particularly those with low magnetic load, i.e. 0.2% and 1% w/w—were corrected for the magnetic signal of the spidroin matrix; then, the *M*_S_ values at *T* = 300 K were compared to that obtained for the magnetite phase in the nanoparticles alone at the same temperature (*M*_S_ at *T* = 300 K was considered to minimize the influence of the low-temperature paramagnetic signal from the spidroin matrix, which, being temperature dependent, was rather difficult to reliably remove from the measured loops).

The thermoremanent magnetization (TRM) was measured following this procedure: the sample was cooled from *T* = 300 K down to *T* = 20 K in an applied magnetic field *H* = 4 kA/m. At *T* = 20 K, the field was removed, and the remanent magnetization was measured as a function of the increasing temperature up to 300 K (heating rate = 3 K/min). The recorded TRM curve was normalized to its initial value.

The Δ*M* plots were built starting from the curves of isothermal remanent magnetization (IRM) and dc demagnetization remanence (DCD), measured at *T* = 20 K using a standard procedure [43]. In particular, in the IRM measurement procedure, an initially demagnetized sample is progressively magnetized by a positive magnetic field increasing from 0 kA/m up to 1.6 × 10^3^ kA/m. The recorded remanence values are plotted as a function of the previously applied magnetic field, and the obtained curve is normalized to its final value. The DCD measurement is similar except that initially the sample is negatively saturated and the curve of remanence vs. *H* is normalized to its initial value (Fig. S16). The Δ*M* plot is obtained by plotting the parameter Δ*M*(H) = DCD(H)-[1- 2 IRM(H)] as a function of *H* (see supplementary section S1).

### Mechanical measurement

All the fibers were tested at room temperature and 25–30% relative humidity (RH) 2 weeks after they were spun. During this period, they were stored in a dry cabinet (Dry Keeper SUNPLATEC) below 20% RH. Single fibers were mounted on paper frames (1 × 1 cm open square window) providing a ~ 1 cm gauge length. The fiber diameter was measured in five randomly picked spots by means of light microscopy and then averaged as previously described. An Instron Single Column 5943 was used to tensile test the artificial silk fibers, with a strain rate of 6 mm/min. The engineering stress was calculated assuming a circular cross-sectional area. From the engineering stress and strain curves, Young’s modulus was obtained from linear regression in the initial elastic part of the curve (by 2% of strain); the strength was obtained as the stress at fracture and the toughness modulus as the area under the curve. The tested fibers were obtained from at least 3–5 different spinning occasions, from which we tested at least 10 fibers. Thus, the mechanical properties reported in this study represent an average of 30–50 fibers. Single one-tail pairwise ANOVA was run with Excel®.

### Light microscopy

Light microscopy inspection was performed using a Nikon Eclipse Ts2R-FL microscope equipped with a DFKNME33UX264 5 MP camera and a CFI Plan Fluor DL-10X objective. The captured images and the diameters were measured with Nikon NIS-Elements BR software.

### Measurement of the density of the fibers

Density measurements of about 35 mg of NT2RepCT fibers were carried out in a Micromeritics® AccuPyc 1330 helium pycnometer (Norcross USA), at 23.0 °C, performing at least 99 measurements.

### Surface plasmon resonance (SPR) assay

To study if there is an interaction between the DMSA-coated nanoparticles and NT2RepCT, we set up an SPR assay using a Biacore 8 K + (Cytiva) and with a Ni^2+^-charged carboxymethylated dextran pre-immobilized with nitrilotriacetic acid (NTA) Sensor S type chip (Cytiva). The instrument was controlled, and the assay was designed with the Biacore 8 K control software version 3.0.12.15655. The immobilization of NT2RepCT on the NTA-coated gold surface was achieved with the 6xHis-tag. We have not chosen covalent coupling methods usually employed to immobilize the ligand in SPR assays due to the pH sensitivity of NT2RepCT.

The temperature of the system was 25 °C throughout the entire experiment. To immobilize, 0.5 M NiCl_2_ (NTA Reagent Kit, Cytiva) was injected with a contact time of 60 s at a flow rate of 10 µL/min, after which the surface was washed with a running buffer containing 3 mM EDTA. Thirty-five micromolar NT2RepCT dimer was captured with a contact time of 300 s also at a flow rate of 10 µL/min. A maximal immobilization level of 8000 RU when the running buffer was HSB-N (Cytiva) or 3000 RU with 20 mM Tris-HCl buffer (pH 8) was reached. On the reference surface, no nickel was injected to not capture the ligand and detect unspecific binding of ligand and analyte.

Analyte solution with a magnetite concentration of 4.5 µg/mL, 0.45 µg/mL, and 0.045 µg/mL was injected over the surface with a contact time of 120 s and a dissociation time of 600 s at 30 µL/min. Regeneration of the chip surface was achieved with 350 mM EDTA (NTA Reagent Kit, Cytiva), using a contact time of 60 s and a flow rate of 30 µL/min.

### Magnetic actuation measurements

The magnetic actuation measurements were done with the same custom-made setup reported by Spizzo et al. [16]. Briefly, the fibers were kept in tension (0.5% strain level) with the support of Agilent Technologies T150 UTM. A commercial set of cubic nickel-plated N42 NdFeB magnets was used to create a non-uniform magnetic field to apply a drag force to the fiber. The intensity of the magnetic field was measured using a FW-Bell 9500 Gaussmeter using a 1-axis Hall probe (the maximum magnetic induction field achieved was *B* = 0.23 T). During the approaching of the magnet, the load on the fiber was recorded with the Agilent technologies T150 UTM. The load recorded was converted in engineering stress assuming the cross-section circular. Every measurement was repeated five times per fiber.

## Supplementary information

Below is the link to the electronic supplementary material.

Supplementary file1 (XLSX 50 KB)

Supplementary file2 (DOCX 6207 KB)

Supplementary file3 (MOV 26733 KB)

Supplementary file4 (MP4 26836 KB)

## Data Availability

All the data necessary to understand the content of this work are reported in the main manuscript, the supplementary material, and additional datasheet files.
