# Supplementary material for: High-performance magnetic artificial silk fibers produced by a scalable and eco-friendly production method
Source: Adv Compos Hybrid Mater. 2024 Oct 2;7(5):163. doi: 10.1007/s42114-024-00962-y (PMC11447077; doi:10.1007/s42114-024-00962-y)
Supplement: Supplementary file 2 — Supplementary file2 (DOCX 6207 KB) [file 42114_2024_962_MOESM2_ESM.docx]

**Supplementary information**

**High-performance magnetic artificial silk fibers produced by a scalable and eco-friendly production method**

Gabriele Greco^1,&^* & Benjamin Schmuck^1,2,&^, Lucia Del Bianco^3^, Federico Spizzo^3^, Luca Fambri^4^, Nicola Maria Pugno^5,6^, Sabino Veintemillas-Verdaguer^7^, Maria Puerto Morales^7^, Anna Rising^1,2^*

^1^ Department of Animal Biosciences, Swedish University of Agricultural Sciences, Box 7011, Uppsala 75007, Sweden

^2^ Department of Biosciences and Nutrition, Karolinska Institutet, Neo, 14183 Huddinge, Sweden

^3^ Department of Physics and Earth Science, University of Ferrara, Via G. Saragat 1, 44122 Ferrara, Italy

^4^ Department of Industrial Engineering and INSTM Research Unit, University of Trento, via Sommarive 9, 38123 Trento, Italy

^5^ Laboratory for Bioinspired, Bionic, Nano, Meta Materials & Mechanics, Department of Civil, Environmental and Mechanical Engineering, University of Trento, Via Mesiano 77, 38123 Trento, Italy

^6^ School of Engineering and Materials Science, Queen Mary University of London, UK, Mile End Road, London E1 4NS, UK

^7^ Instituto de Ciencia de Materiales de Madrid, ICMM/CSIC, Sor Juana Inés de la Cruz 3, 28049 Madrid, Spain

^&^ these authors contributed equally

*Corresponding authors: [gabriele.greco@slu.se](mailto:gabriele.greco@slu.se); [anna.rising@slu.se](mailto:anna.rising@slu.se);


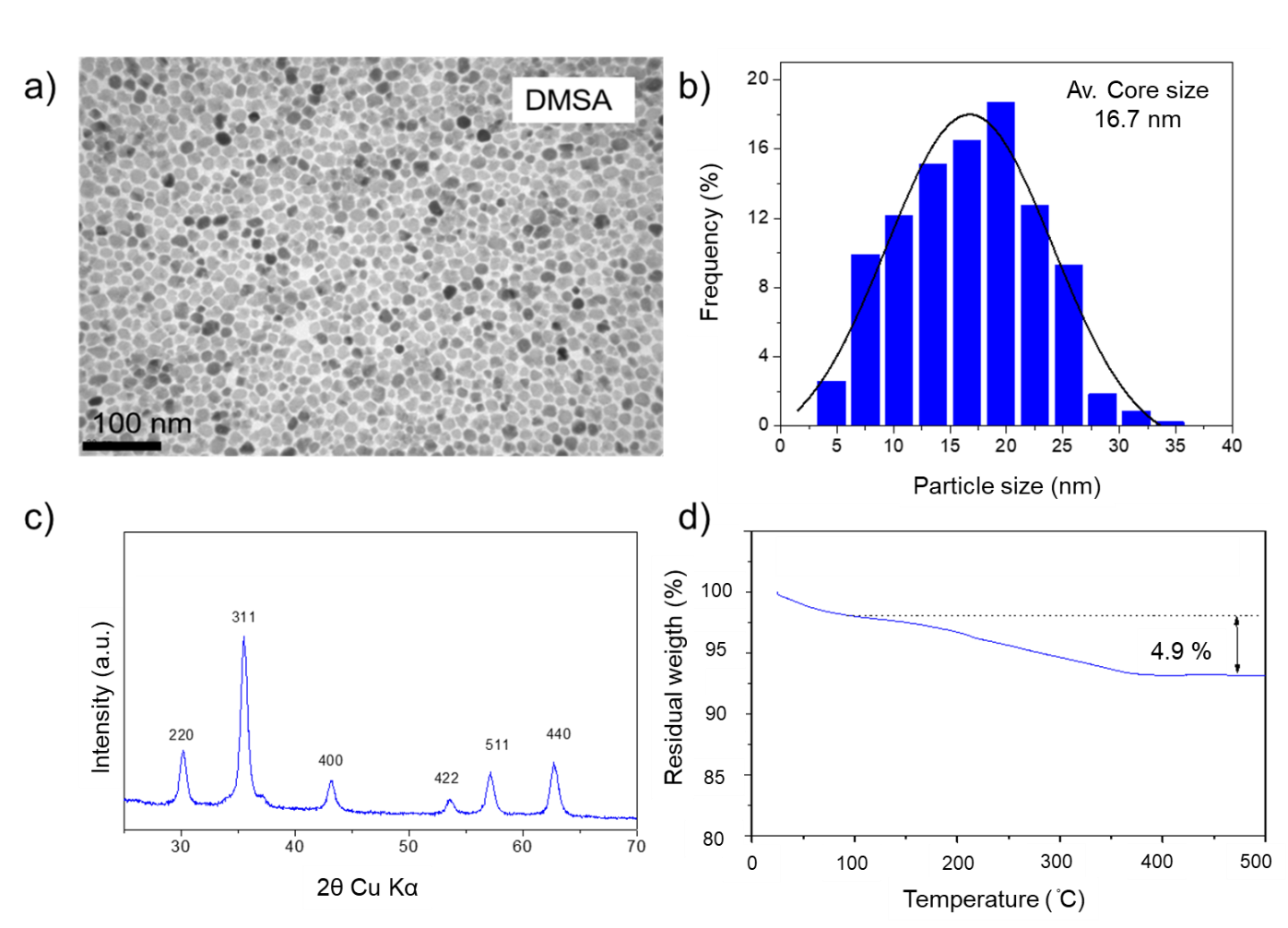


Figure S1: a) TEM image of the DMSA-coated magnetite nanoparticles, b) nanoparticle core size distribution, obtained by measuring about 800 particles, c) X-ray diffraction and d) thermogravimetric analysis.

**
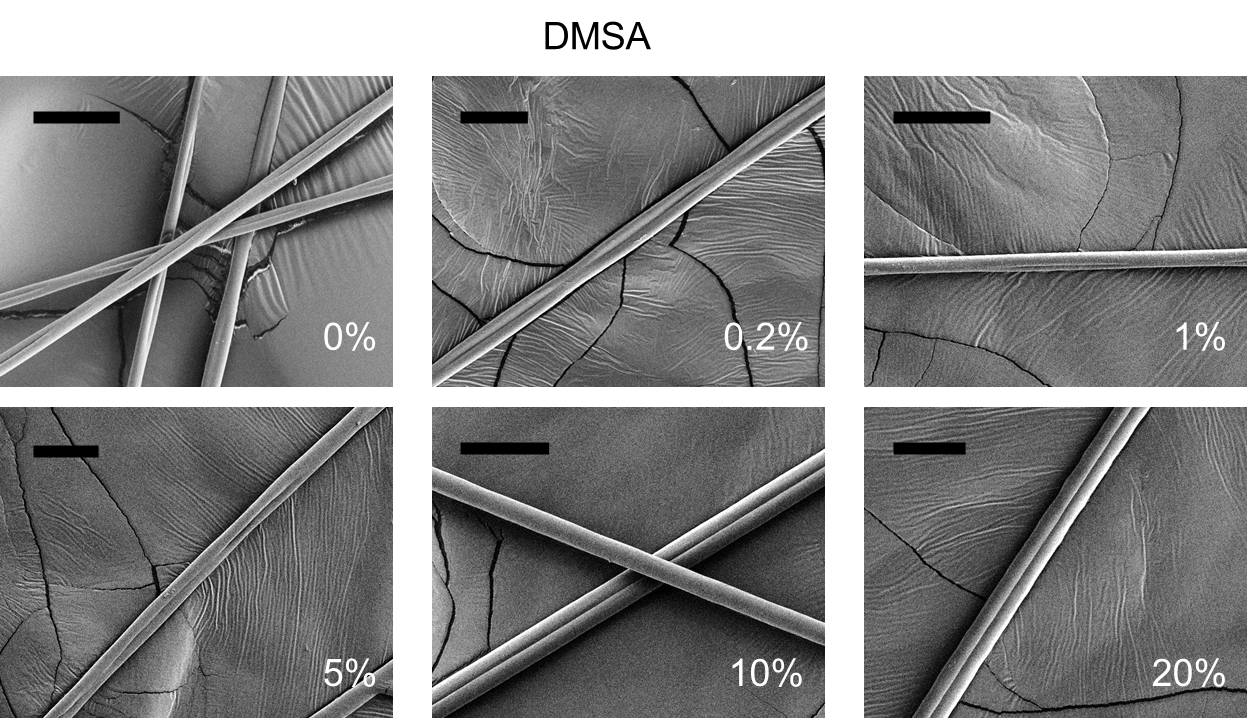
**

Figure S2: SEM images of the artificial magnetic spider silk fibers with different nominal w/w magnetite concentrations. Scale bars are 20 μm.


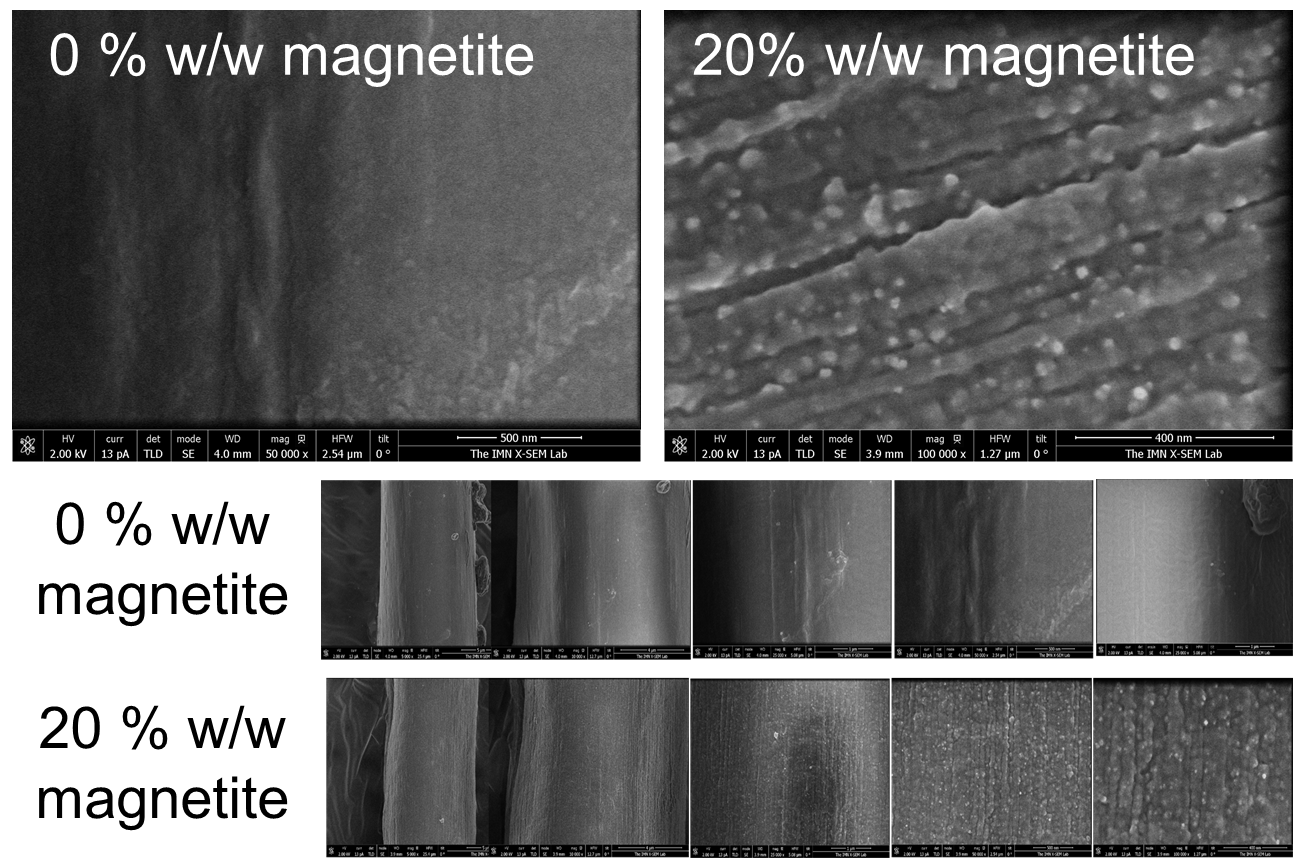


Figure S3: Representative high magnification SEM images of the NT2RepCT fibers (i.e., fibers containing no nanoparticles) and 20% w/w (nominal) of magnetite, which indicates homogeneous dispersion of the nanoparticles.


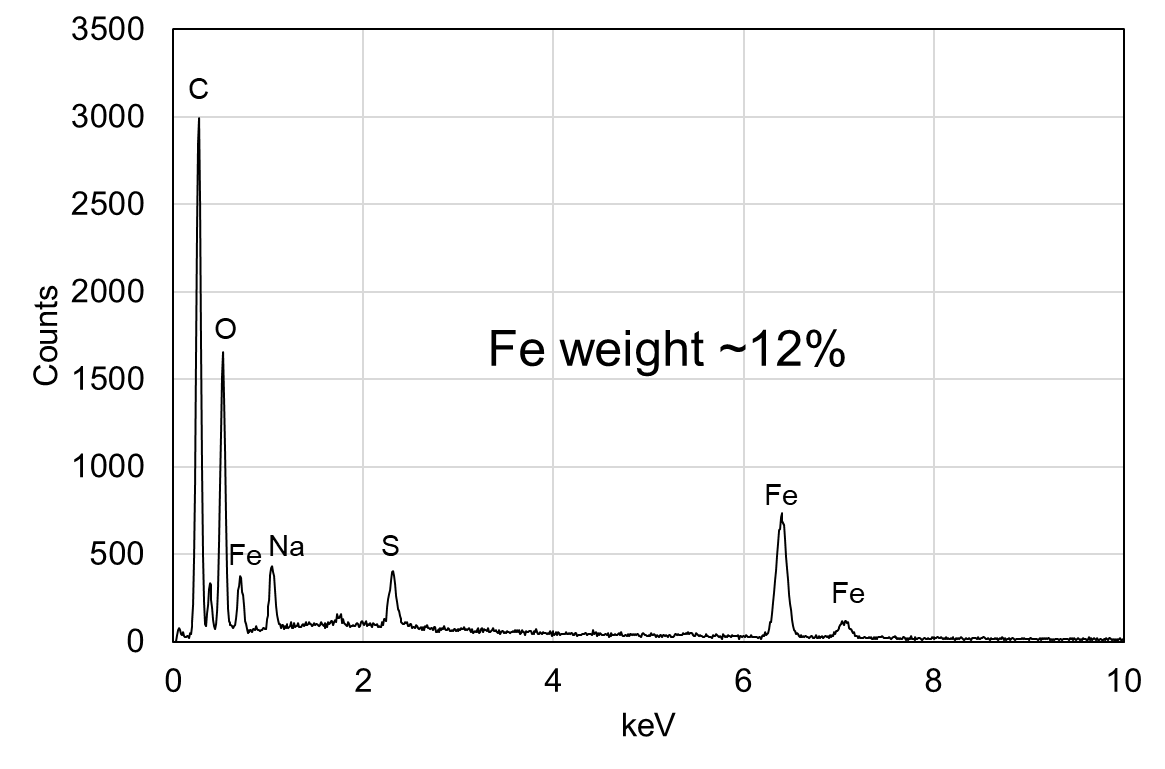


Figure S4: EDX spectra of the fibers having a 20% w/w nominal concentration of magnetite. According to the spectra, these fibers contain 12% w/w Fe, which corresponds to ~17% w/w magnetite.

Supplementary section S1: Magnetic properties of the fibers

For brevity, in this section and in Table S3, the composite fibers have been labeled as DX, where D stands for DMSA and X is a number referring to the nominal magnetite concentration. Magnetic loops, i.e. curves of magnetization M vs. magnetic field H, were measured on the whole set of composite fibers and on the nanoparticles alone by SQUID magnetometer, at T = 5 K and 300 K. For the nanoparticles alone (Fig. S5), the magnetization at H = 4×10^3^ kA/m (taken as the saturation magnetization M_S_) was corrected for the presence of the non-magnetic DMSA coating (~ 5 % w/w) in order to estimate the M_S_ of the magnetite phase alone. Hence, the obtained values were M_S_ = (71.3 ± 0.7) Am^2^/kg at 5 K and M_S_ = (62.8 ± 0.6) Am^2^/kg at 300 K.

Figure S5: Magnetic loops measured at T = 5 K and 300 K on the DMSA-coated nanoparticles (i.e. the nanoparticles alone); the inset is a close-up of the central region of the loops.

The loops measured on the fiber samples at T = 5 K and 300 K are shown in Fig. S6 and Fig. 3, respectively. The values of saturation magnetization M_S_ at the two temperatures are reported in Table S3 together with the weight fractions of magnetite in the fibers (calculated by comparing the values of M_S_ to that obtained for the magnetite in the nanoparticles alone). The estimated concentrations are in good agreement with the nominal ones.

At T = 5 K, the coercivity H_C_ of samples D02, D1, and D5 is in the 27.1 ÷ 27.8 kA/m range, i.e. equal within the experimental errors; H_C_ for D10 and D20 is slightly lower, 25.5 ÷ 25.7 kA/m. At T = 300 K, no magnetic hysteresis is observed either in the nanoparticles alone or in the fiber samples, i.e. H_C_ and the remanent magnetization are null (Fig. 3a). This is consistent with a superparamagnetic behavior of the magnetic moments of the nanoparticles, in the adopted experimental conditions. In fact, it is known that the magnetic moment of an isolated single-domain nanoparticle can undergo magnetic relaxation, possibly culminating in the superparamagnetic behavior when the thermal energy is comparable to the anisotropy energy barrier for its reversal[1]. The blocking temperature T_B_, above which a magnetic nanoparticle enters the superparamagnetic regime, is given by the relation:

$T_{B}=KV/[k_{B}\ln(t_{m}f_{0})]$ (1)

where KV is the anisotropy energy barrier (K magnetic anisotropy coefficient, V volume of the nanoparticle), k_B_ is the Boltzmann constant, t_m_ is the measuring time characteristic of the adopted analysis technique and *f*_0_ is a frequency factor[1]. For SQUID measurements, t_m_ and *f*_0_ are usually taken equal to 100 s and 10^9^ s^-1^, respectively, and therefore ln(t_m_*f*_0_) ~ 25[2,3].

Figure S6: Left: Magnetic hysteresis loops measured at T = 5 K on the set of composite fibers, corrected for the magnetic signal from the spidroin matrix. Right: close-up of the central region of the loops. Different colors indicate different nominal w/w magnetite concentrations.

Information on the relaxing behavior of the nanoparticles in the fibers was gained through the analysis of the thermoremanent magnetization (TRM) vs. T and corresponding temperature derivatives curve, i.e. [-d(TRM)/dT] vs. T[1,4,5]. The latter provides a figure of the distribution of anisotropy energy barriers associated with the assembly of nanoparticles embedded in the fibers and, based on equation (1), also of the distribution of blocking temperatures T_B_. The results are shown in Fig. 3b,c. In all samples, TRM goes to zero with increasing temperature, confirming the progressive entrance of the nanoparticles into the superparamagnetic regime. For samples D02, D1, and D5, the derivative curves exhibit a similar profile, consistent with a quite narrow anisotropy energy barrier distribution with a peak temperature of 70-80 K (Fig. 3c), which can be taken as a mean blocking temperature <T_B_>. From equation (1), considering that the mean size of the magnetic core of the nanoparticles is ~ 17 nm (Table S1) and setting K equal to that of bulk magnetite (1.0×10^4^ J/m^3^), a value <T_B_> ~ 72 K is obtained. This good agreement between the measured and predicted average blocking temperature indicates that, in the samples with low magnetic load, the nanoparticles are very homogeneously dispersed and are far apart from each other, so that dipolar magnetic interactions are not strong enough to affect their relaxing behavior. A slightly different situation is observed for samples D10 and D20, which show broader distributions reaching a maximum at ~100 K and ~110 K, respectively (Fig. 3c). The shift to a higher temperature of the anisotropy energy barrier distribution is to be ascribed to the existence of non-negligible interparticle dipolar interactions[1–3,6–9]. Since dipolar interactions are demagnetizing in nature, their presence can also account for the smaller H_C_ of D10 and D20 at T = 5 K, compared to the other samples (Table S3). To evaluate to what extent the magnetic behavior of the nanoparticles is affected by dipolar interactions, a mean effective magnetic anisotropy <K_eff_> can be calculated from equation (1), taking the peak temperature of the distribution as the <T_B_> value. The obtained results are <K_eff_> = 1.4×10^4^ J/m^3^ for D10 and <K_eff_> = 1.5×10^4^ J/m^3^ for D20, significantly larger than that of bulk magnetite. However, they are very similar, which is unexpected if one considers that, with doubling the fraction of nanoparticles, the interparticle distance should reduce and therefore the strength of dipolar interactions should increase. Indeed, these findings reveal that a less homogeneous spatial distribution of the nanoparticles is attained in D10 and D20, compared to the fibers with lower magnetic load. The existence of regions with different local concentrations of nanoparticles in the spidroin matrix can explain the observed broadening of the distribution of anisotropy energy barriers (i.e., of effective barriers). On the other hand, an inhomogeneous arrangement of the nanoparticles may also imply that on average the interparticle distance is similar in the two samples, thus accounting for the close values of <K_eff_>.

This description is supported by the analysis of the ΔM plots, which is a powerful method to gain information on the nature of magnetic interactions in nanoparticle assemblies [2,3,5]. In particular, a negative ΔM plot indicates the existence of dipolar interactions and the absolute peak value of ΔM provides qualitative information on their strength. Hence, the ΔM plots collected on the composite fibers at T = 20 K, shown in Fig. S7, confirm the existence of dipolar interactions whose strength tends to increase with increasing the nanoparticle load, but is substantially similar in samples D10 and D20.

Figure S7: ΔM-plots at T = 20 K for the composite fibers. Different colors indicate different nominal w/w magnetite concentrations. The ΔM parameter is calculated as ΔM(H) = DCD(H)-[1- 2IRM(H)].


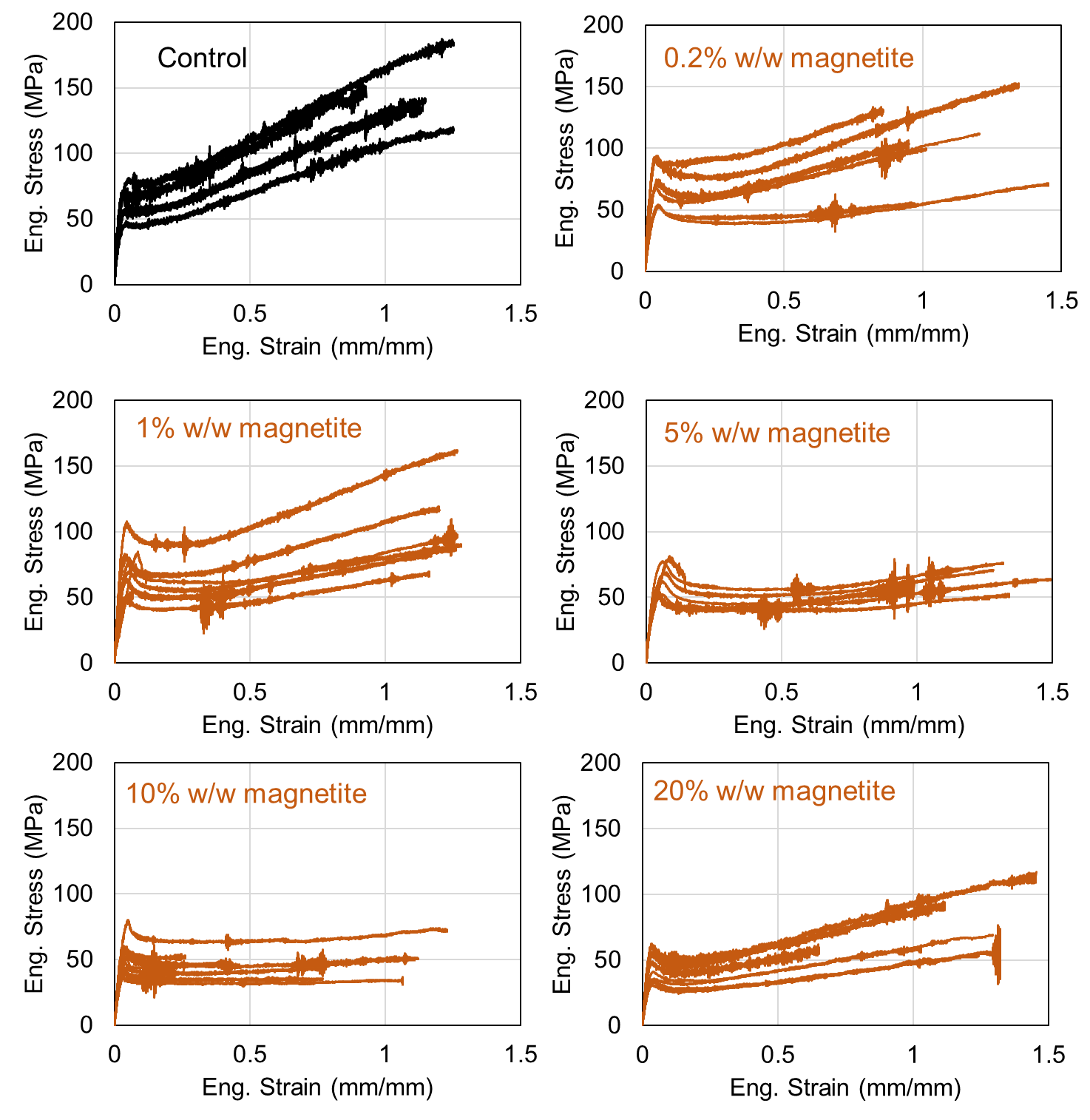


Figure S8: Representative stress-strain curves of composite fibers with different levels of nominal w/w magnetite concentrations.


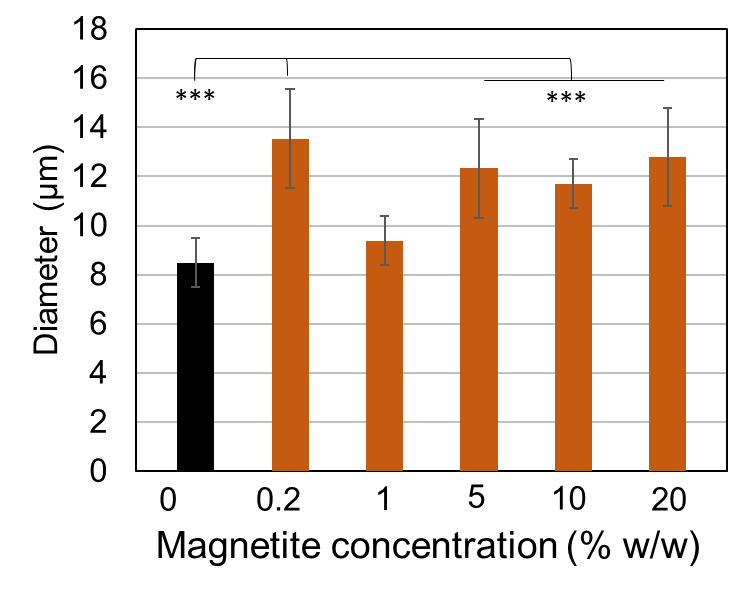


Figure S9: Diameters of the magnetic artificial silk fibers vs. the nominal magnetite concentration (w/w). * Indicate p-value <0.05, ** p-value <0.01, and *** p-value < 0.001.


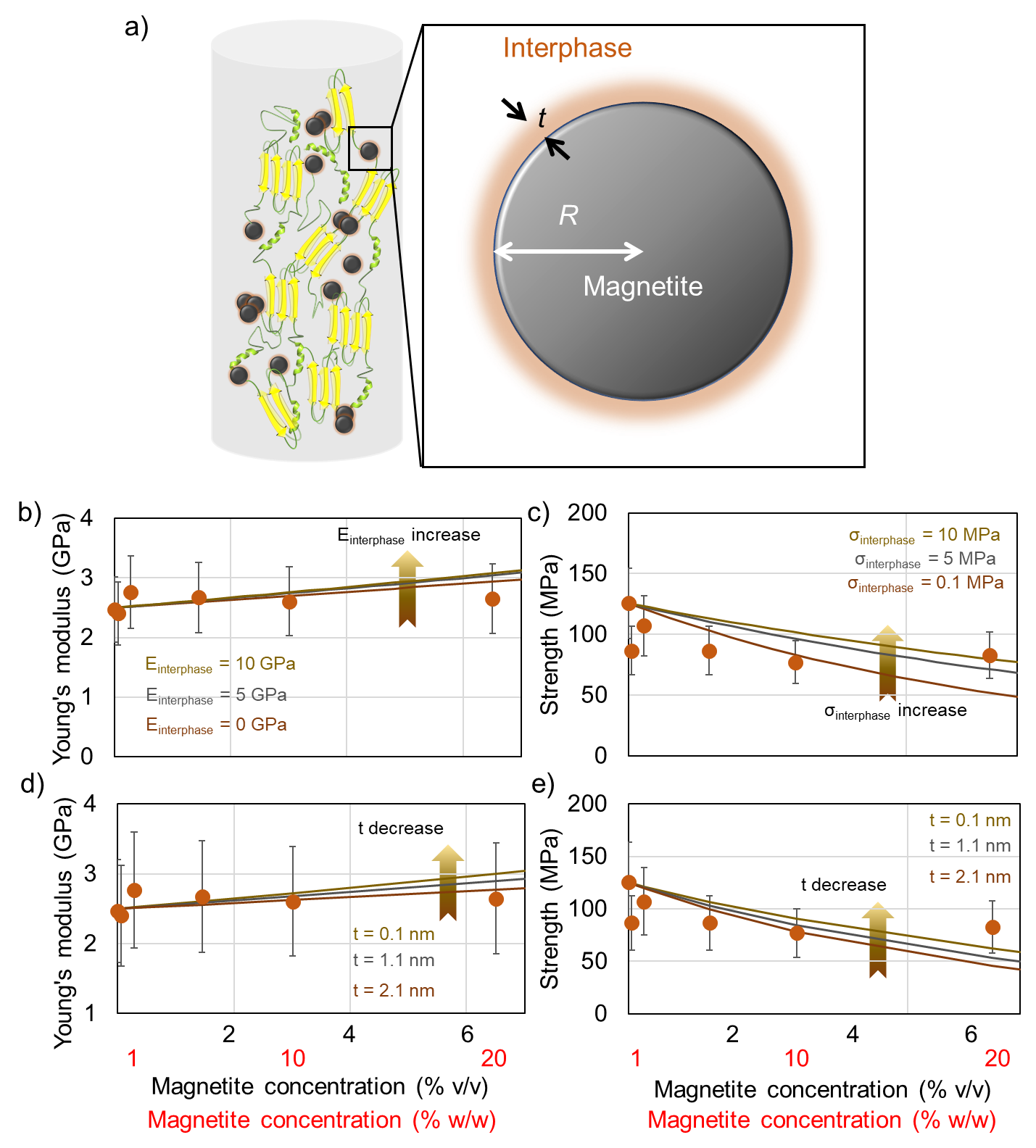


Figure S10: a) Schematic of the model used to interpret the impact of the mechanical properties of the interphase on the mechanical properties of the composite fibers. The relative dimensions between the nanoparticles and the secondary structure is only for representation purposes. Plot of the experimental values of the mechanical properties and the theoretical lines obtained from the model, and in particular for equation b) 2, c) 3, d), 4, and e) 5. To plot the model, the protein matrix (NT2RepCT) has been considered with a strength of 125 MPa and a Young’s modulus of 2.5 GPa. For panel d), a value of E_interphase_ = 0 GPa was used. For panel e), a value of σ_interphase_ = 0.1 MPa was used.

Supplementary section S2: Analytical model of the composite

The analytical model to explain the effect of the nanoparticle volumetric concentration on the mechanical properties of the magnetic artificial silk fiber was developed by Zare[10], which is an extension of Maxwell[11] and Pukànsky[12] theories for composites (for Young’s modulus *E* and strength *σ_C_* of the composite respectively). Fig. S10a depicts the geometry of the system: thickness of the interphase (*t*), and radius of the nanoparticle (*R*). In this model, we assumed that the nanoparticles are homogeneously dispersed in the matrix, which agrees with the experimental findings. Furthermore, we consider the properties of the interphase as a combination of the DMSA coating mechanical properties, the interface interactions between the magnetite and the DMSA, and the interface interactions between the DMSA and the protein.

In the model is added a third phase (the interphase between the filler and the matrix). The equations for Young’s modulus of the fibrous composite and the strength are

$$E=E_{m}\frac{1+\frac{2\phi_{f}\left( E_{f}/E_{m}-1 \right)}{E_{f}/E_{m}+2}+\frac{2\phi_{i}\left( E_{i}/E_{m}-1 \right)}{E_{i}/E_{m}+2}}{1-\frac{\phi_{f}\left( E_{f}/E_{m}-1 \right)}{E_{f}/E_{m}+2}-\frac{\phi_{i}\left( E_{i}/E_{m}-1 \right)}{E_{i}/E_{m}+2}} (2)$$

$$\sigma_{R}=\frac{\sigma_{c}}{\sigma_{m}}=\frac{1-\phi_{f}}{1+2.5\phi_{f}}\exp\left[ \left( \phi_{f}+3\phi_{f}\left( \frac{\phi_{i}}{\phi_{f}}+1 \right)^{\frac{1}{3}}-3\phi_{f} \right)\ln\left( \frac{\sigma_{i}}{\sigma_{m}} \right) \right] (3)$$

where *E_m_*, *E_i_*, and *E_f_* are the Young’s moduli of the matrix, the interphase and the filler, *ϕ_f_* and *ϕ_i_* are the volumetric fractions of the filler and the interphase respectively, and finally *σ_R_*, *σ_c_, σ_m_*, and *σ_i_* are the relative strength of the composite, the strength of the composite, the matrix, and the interphase respectively.

Equations 2 and 3 can be also written in terms of *t* and *R*. They become

$$E=E_{m}\frac{1+\frac{2\phi_{f}\left( E_{f}/E_{m}-1 \right)}{E_{f}/E_{m}+2}+\frac{2\phi_{f}\left[ \left( \frac{R+t}{R} \right)^{3}-1 \right]\left( E_{i}/E_{m}-1 \right)}{E_{i}/E_{m}+2}}{1-\frac{\phi_{f}\left( E_{f}/E_{m}-1 \right)}{E_{f}/E_{m}+2}-\frac{\phi_{f}\left[ \left( \frac{R+t}{R} \right)^{3}-1 \right]\left( E_{i}/E_{m}-1 \right)}{E_{i}/E_{m}+2}} (4)$$

$$\sigma_{R}=\frac{\sigma_{c}}{\sigma_{m}}=\frac{1-\phi_{f}}{1+2.5\phi_{f}}\exp\left[ \left( \phi_{f}+3\phi_{f}\left( \frac{\phi_{f}\left[ \left( \frac{R+t}{R} \right)^{3}-1 \right]}{\phi_{f}}+1 \right)^{\frac{1}{3}}-3\phi_{f} \right)\ln\left( \frac{\sigma_{i}}{\sigma_{m}} \right) \right] (5)$$

To plot equations 2-5, we calculated the volume fractions of magnetite (Table S4) considering the mass density values of bulk magnetite (~ 5 x 10^3^ kg/m^3^), DMSA (~ 1.6 x 10^3^ kg/m^3^, see ref[13]), and artificial spider silk fibers (measured value [1.41 ± 0.09] x 10^3^ kg/m^3^, Fig. S11). The volumetric fraction of the interphase is here assumed to be the volumetric fraction of the DMSA coating. Furthermore, we also considered that the DMSA coating constitutes ~5% w/w of the nanoparticle (Fig. S1). This data is important to estimate the thickness of the interphase, i.e., the thickness of the DMSA coating, which can be obtained by geometrical considerations and turns out to be ~ 0.4 nm. To plot the graph, we used values between 0.1-2.1 nm to show a broader range. Furthermore, the parameters that can be extrapolated from experimental data are the radius of the magnetite nanoparticle (~9 nm including DMSA coating), the strength and Young’s modulus of the matrix (125 MPa and 2.5 GPa from the mechanical data obtained with tensile tests) and Young’s modulus of the magnetite (300 GPa from literature[14]). The unknown parameters are the strength and Young’s modulus of the interphase. We thus used different numbers to show their effect on the mechanical properties of the composite fiber. Once these parameters are defined, the strength and Young’s modulus of the composite fiber can be plotted vs. volumetric magnetite fraction (Fig. S10). From this model and in the conditions that we explored to fit the graphs, it is possible to state that, to have a significant reinforcement, the strength and Young’s modulus of the interphase have to be much higher compared to the matrix. In particular, to produce a significant increase of Young’s modulus at low magnetite concentrations the interphase must have a Young’ modulus >>10 GPa (Fig. S10b). To lead to an increase in strength, the strength of the interphase must be at least >125 MPa (equation 3, Fig. S10c). Moreover, the thickness of the DMSA coating seems to have a minor effect in the explored range and at volumetric magnetite concentrations lower than 20% (Fig. S10d, e). This also is consistent with the estimation of the DMSA coating thickness (0.4 nm).


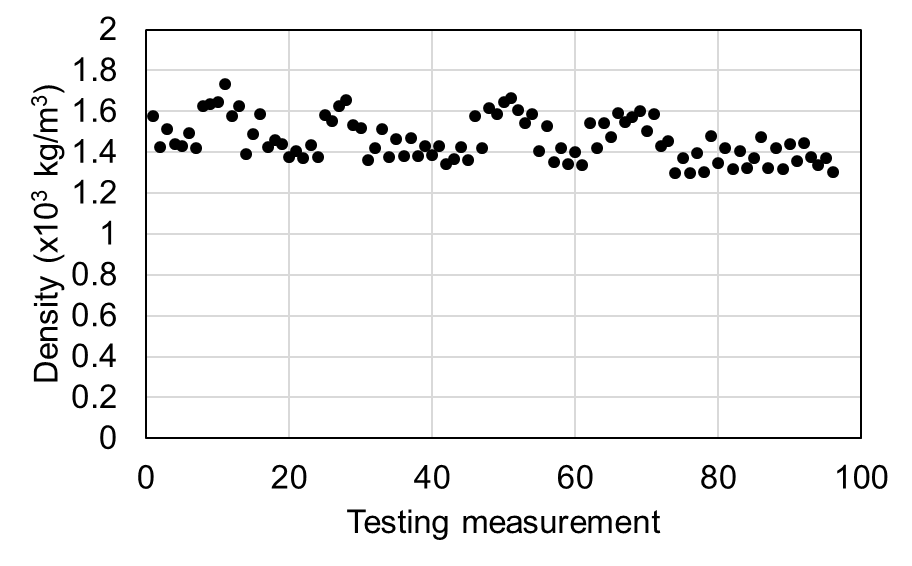


*Figure S11: Density measurements of about 35 mg of NT2RepCT fibers carried out in a pycnometer at 23.0°C. The obtained value of density is (1.41 ± 0.09) x 10^3^ kg/m^3^.*


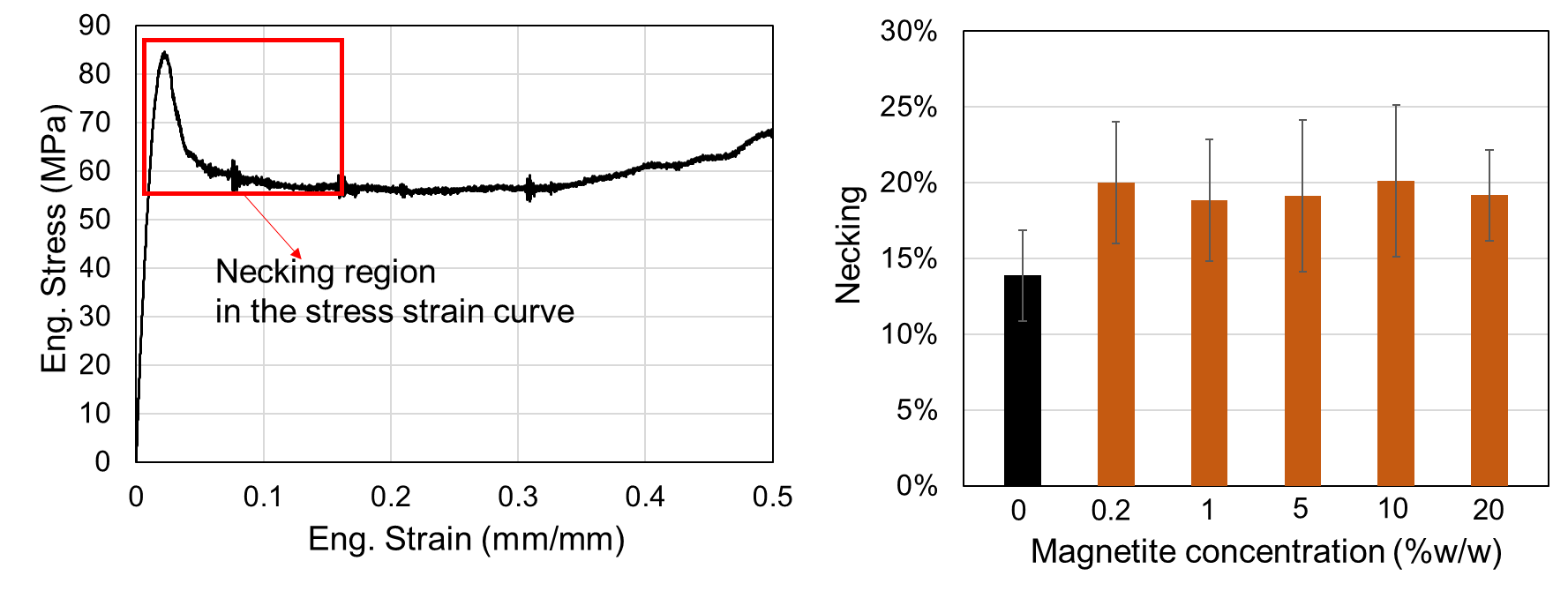


Figure S12: Representative necking of a representative stress strain curve of a silk fiber and necking values vs. the nominal magnetite concentration (w/w).

Supplementary section S3: Surface Plasmon Resonance (SPR)-assay

The basic principle of an SPR assay is that the reflection angle of a light beam away from a gold surface is influenced by the refractive index of the surface, which is directly correlated with the weight of the molecules on a gold chip. Thus, the presence of additional weight on a gold surface can be detected label-free, by directing a polarized light beam with a single wavelength onto the bottom of the gold surface.

Since immobilization of the ligand, NT2RepCT, on the gold surface of the sensor chip increased the weight, an increase in the relative response was detected (Fig. S13 A). In fact, an immobilization level of more than 3000 RU was achieved, which is close to the upper limit of this chip, also indicated by the fact that a substantial amount of NT2RepCT leaks from the surface after the injection event (indicated by an arrow). To detect the presence or absence of an interaction between the spidroin and the magnetite nanoparticles, the nanoparticles are subsequently injected over the same surface, which is available to be captured by the NT2RepCT. If both entities interact, this will add an additional weight to the gold surface, which should increase the response relative to a reference surface where no ligand is bound. The sensorgram shown in Fig. S13 is reference subtracted (on the reference surface NT2RepCT was not immobilized) and shows the point where the analyte (the nanoparticles) was injected (Fig. S13B). Even though a very high immobilization level of NT2RepCT and a high concentration of nanoparticles (4.6 µg/mL) was used in this particular assay, the instrument did not detect any additional weight on the sensor surface compared to the reference and the blank sample. This indicates that NT2RepCT and the nanoparticles do not interact or the interaction is very weak.


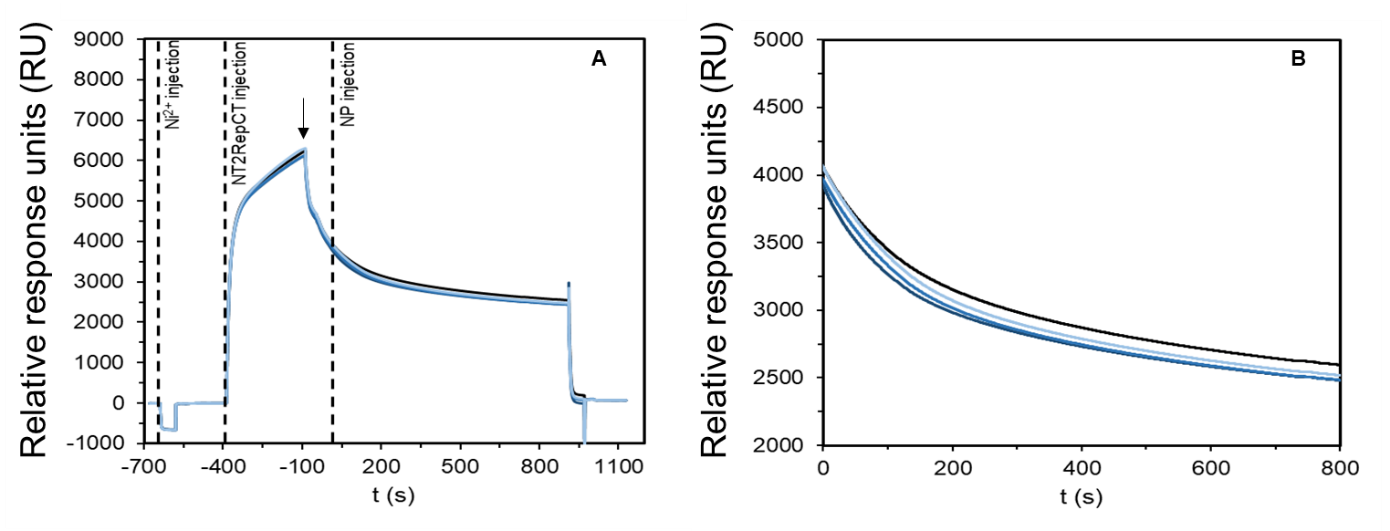


*Figure S13: Sensorgram obtained with an SPR-assay to detect if there is an interaction between the DMSA-coated magnetite nanoparticles and NT2RepCT. (A) Complete sensorgram featuring the events of nickel binding to an NTA-chip, followed by washing and injection of the ligand (NT2RepCT), and finally injection of the nanoparticles. These events are indicated by dashed lines. (B) Zoom in on the sensorgram shown in A. Injection of Analyte (DMSA coated nanoparticles) onto NT2RepCT immobilized on a gold chip in an SPR-assay. The injection event of 4.6 µg/ml (dark blue), 0.46 µg/ml (blue), 0.046 µg/ml (light blue), and 0 nM (black) magnetite starts at t=0s.*


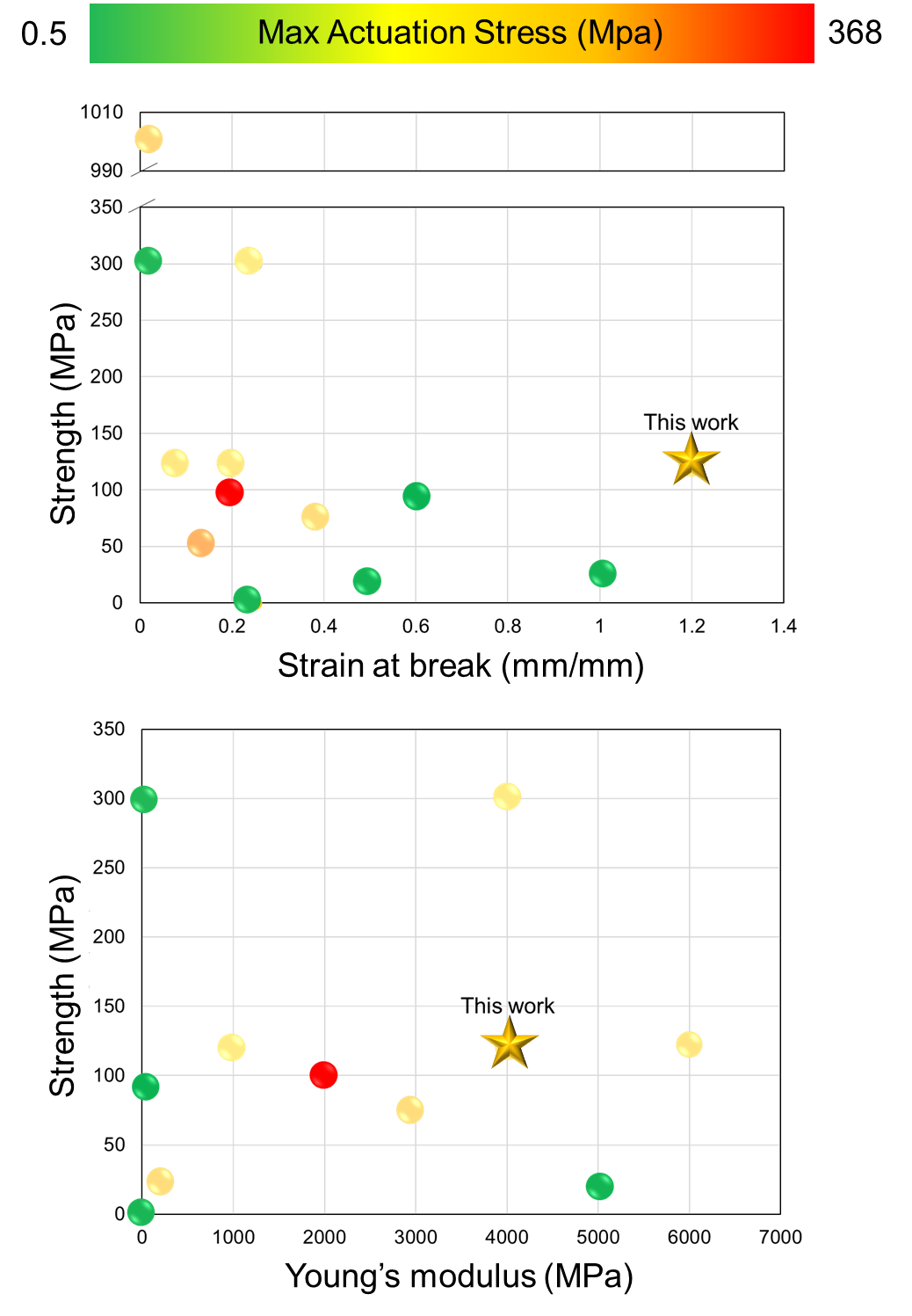


Figure S14: Mechanical properties plotted vs. the maximal actuation stress generated by standard fibrous materials, the data were obtained from[15–28].

*Figure S15: Curves of Magnetization M vs. magnetic field H measured on the NT2RepCT fibers (i.e., fibers containing no nanoparticles) at the indicated temperatures.*

*Figure S16: Isothermal remanent magnetization (IRM) and dc demagnetization remanence (DCD) curves, measured at T = 20 K on fibers with 20% w/w magnetite.*

Table S1: Physical properties of the DMSA-coated magnetite nanoparticles.

| Core | Coating | Average nanoparticle core size  TEM (nm) | Crystal size X-ray (nm) | Hydrodynamic size  (nm) | Z-Potential  (mV) |
| --- | --- | --- | --- | --- | --- |
| Fe_3_O_4_ | Dimercaptosuccinic acid | 16.7 (σ=0.16) | ~13 | 74(PDI= 0.23) | -27 |

Table S2: The nanomaterial concentrations that were used to achieve the optimal mechanical properties of different silk-nanomaterials composites.

| ***Type of nanomaterial*** | ***Concentration to obtain maximum strain at break or strength (w/w)*** | ***Max concentration explored (w/w)*** | ***Reference*** |
| --- | --- | --- | --- |
| *Single-walled carbon nanotubes* | *1%* | *2%* | [29] |
| *Multiwalled carbon nanotubes* | *1%* | *1.5%* | [30] |
| *Carbon nanotubes* | *<0.5%* | *<0.5%* | [31] |
| *Carbon nanotubes* | *0.2%* | *1%* | [32] |
| *Single-walled carbon nanotubes* | *0.2%* | *1%* | [33] |
| *Graphene oxide* | *0.1%* | *0.2%* | [34] |
| *Magnetite nanoparticles* | *0.2-1%* | *20%* | *This study* |

*Table S3: Magnetization and magnetic coercivity of the artificial silk fibers and estimated w/w magnetite concentrations.*

| **Sample** | **Nominal magnetite fraction**  **(w/w %)** | **M_S_**  **at T = 5 K**  **(Am^2^/kg)**  **± 3%** | **M_S_**  **at T = 300 K**  **(Am^2^/kg)**  **± 3%** | **H_C_**  **at T = 5K**  **(kA/m )**  **± 0.4** | **Estimated magnetite fraction**  **(w/w %)**  **± 5%** |
| --- | --- | --- | --- | --- | --- |
| D02 | 0.2 | 0.172 | 0.148 | 27.1 | 0.24 |
| D1 | 1 | 0.88 | 0.74 | 27.1 | 1.18 |
| D5 | 5 | 4.1 | 3.50 | 27.8 | 5.6 |
| D10 | 10 | 6.5 | 5.7 | 25.5 | 9.1 |
| D20 | 20 | 14.3 | 12.5 | 25.7 | 20 |

Table S4: Estimated magnetite volume fractions in the magnetic artificial silk fibers.

| **Nominal magnetite fraction**  **(w/w %)** | **Estimated magnetite fraction (% volume)**  **± 10%** |
| --- | --- |
| 0.2 | 0.07 |
| 1 | 0.33 |
| 5 | 1.63 |
| 10 | 2.7 |
| 20 | 6.5 |

**Additional References:**

[1] J.L. Dormann, D. Fiorani, E. Tronc, Magnetic Relaxation in Fine-Particle Systems, in: Adv. Chem. Phys. Vol. XCVIII, John Wiley & Sons, Inc.: New York, USA, 2007: pp. 283–494. https://doi.org/10.1002/9780470141571.ch4.

[2] L. Del Bianco, F. Spizzo, G. Barucca, M.R. Ruggiero, S. Geninatti Crich, M. Forzan, E. Sieni, P. Sgarbossa, Mechanism of magnetic heating in Mn-doped magnetite nanoparticles and the role of intertwined structural and magnetic properties, Nanoscale. 11 (2019) 10896–10910. https://doi.org/10.1039/c9nr03131f.

[3] M. Blanco-Mantecón, K. O’Grady, Interaction and size effects in magnetic nanoparticles, J. Magn. Magn. Mater. 296 (2006) 124–133. https://doi.org/10.1016/j.jmmm.2004.11.580.

[4] J.G. Ovejero, F. Spizzo, M.P. Morales, L. Del Bianco, Mixing iron oxide nanoparticles with different shape and size for tunable magneto-heating performance, Nanoscale. 13 (2021) 5714–5729. https://doi.org/10.1039/d0nr09121a.

[5] K. O’Grady, R.W. Chantrell, Remanence Curves of Fine Particle Systems I: Experimental Studies, in: Magn. Prop. Fine Part., Elsevier, 1992: pp. 93–102. https://doi.org/10.1016/b978-0-444-89552-3.50017-7.

[6] G.F. Goya, T.S. Berquó, F.C. Fonseca, M.P. Morales, Static and dynamic magnetic properties of spherical magnetite nanoparticles, J. Appl. Phys. 94 (2003) 3520–3528. https://doi.org/10.1063/1.1599959.

[7] M. El-Hilo, K. O’Grady, R.W. Chantrell, Susceptibility phenomena in a fine particle system. I. Concentration dependence of the peak, J. Magn. Magn. Mater. 114 (1992) 295–306. https://doi.org/10.1016/0304-8853(92)90272-P.

[8] C. Binns, M.J. Maher, Q.A. Pankhurst, D. Kechrakos, K.N. Trohidou, Magnetic behavior of nanostructured films assembled from preformed Fe clusters embedded in Ag, Phys. Rev. B - Condens. Matter Mater. Phys. 66 (2002) 1–12. https://doi.org/10.1103/PhysRevB.66.184413.

[9] J.G. Ovejero, F. Spizzo, M.P. Morales, L. Del Bianco, Nanoparticles for magnetic heating: When two (or more) is better than one, Materials (Basel). 14 (2021) 6416. https://doi.org/10.3390/ma14216416.

[10] Y. Zare, The roles of nanoparticles accumulation and interphase properties in properties of polymer particulate nanocomposites by a multi-step methodology, Compos. Part A Appl. Sci. Manuf. 91 (2016) 127–132. https://doi.org/10.1016/j.compositesa.2016.10.003.

[11] J.C. Maxwell, A Treatise on Electricity and Magnetism, Cambridge University Press, 1873. https://doi.org/10.1017/CBO9780511709333.

[12] B. Pukánszky, Influence of interface interaction on the ultimate tensile properties of polymer composites, Composites. 21 (1990) 255–262. https://doi.org/10.1016/0010-4361(90)90240-W.

[13] Chem-Search engine, (2023).

[14] D. Chicot, J. Mendoza, A. Zaoui, G. Louis, V. Lepingle, F. Roudet, J. Lesage, Mechanical properties of magnetite (Fe3O4), hematite (Fe2O3) and goethite (FeOOH) by instrumented indentation and molecular dynamics analysis, Mater. Chem. Phys. 129 (2011) 862–870. https://doi.org/10.1016/j.matchemphys.2011.05.056.

[15] J.A. Lee, Y.T. Kim, G.M. Spinks, D. Suh, X. Lepró, M.D. Lima, R.H. Baughman, S.J. Kim, All-solid-state carbon nanotube torsional and tensile artificial muscles, Nano Lett. 14 (2014) 2664–2669. https://doi.org/10.1021/nl500526r.

[16] W. Guo, C. Liu, F. Zhao, X. Sun, Z. Yang, T. Chen, X. Chen, L. Qiu, X. Hu, H. Peng, A novel electromechanical actuation mechanism of a carbon nanotube fiber, Adv. Mater. 24 (2012) 5379–5384. https://doi.org/10.1002/adma.201201845.

[17] J. Mu, M.J. de Andrade, S. Fang, X. Wang, E. Gao, N. Li, S.H. Kim, H. Wang, C. Hou, Q. Zhang, M. Zhu, D. Qian, H. Lu, D. Kongahage, S. Talebian, J. Foroughi, G. Spinks, H. Kim, T.H. Ware, H.J. Sim, D.Y. Lee, Y. Jang, S.J. Kim, R.H. Baughman, Sheath-run artificial muscles, Science (80-. ). 155 (2019) 150–155. https://doi.org/10.1126/science.aaw2403.

[18] W. Wang, C. Xiang, Q. Liu, M. Li, W. Zhong, K. Yan, D. Wang, Natural alginate fiber-based actuator driven by water or moisture for energy harvesting and smart controller applications, J. Mater. Chem. A. 6 (2018) 22599–22608. https://doi.org/10.1039/c8ta08064j.

[19] D.W. Lee, S.H. Kim, M.E. Kozlov, X. Lepró, R.H. Baughman, S.J. Kim, Magnetic torsional actuation of carbon nanotube yarn artificial muscle, RSC Adv. 8 (2018) 17421–17425. https://doi.org/10.1039/c8ra01040d.

[20] Y. Kim, H. Yuk, R. Zhao, S.A. Chester, X. Zhao, Printing ferromagnetic domains for untethered fast-transforming soft materials, Nature. 558 (2018) 274–291. https://doi.org/10.1038/s41586-018-0185-0.

[21] A. Maziz, A. Concas, A. Khaldi, J. Stålhand, N.K. Persson, E.W.H. Jager, Knitting and weaving artificial muscles, Sci. Adv. 3 (2017) 1–12. https://doi.org/10.1126/sciadv.1600327.

[22] L. Ionov, G. Stoychev, D. Jehnichen, J.U. Sommer, Reversibly Actuating Solid Janus Polymeric Fibers, ACS Appl. Mater. Interfaces. 9 (2017) 4873–4881. https://doi.org/10.1021/acsami.6b13084.

[23] C.S. Haines, M.D. Lima, N. Li, G.M. Spinks, J. Foroughi, J.D.W. Madden, S.H. Kim, S. Fang, M.J. De Andrade, F. Göktepe, Ö. Göktepe, S.M. Mirvakili, S. Naficy, X. Lepró, J. Oh, M.E. Kozlov, S.J. Kim, X. Xu, B.J. Swedlove, G.G. Wallace, R.H. Baughman, Artificial muscles from fishing line and sewing thread, Science (80-. ). 343 (2014) 868–872. https://doi.org/10.1126/science.1246906.

[24] J. Park, J.W. Yoo, H.W. Seo, Y. Lee, Electrically controllable twisted-coiled artificial muscle actuators using surface-modified polyester fibers, Smart Mater. Struct. 26 (2017). https://doi.org/10.1088/1361-665X/aa5323.

[25] M.D. Lima, N. Li, M.J. De Andrade, S. Fang, J. Oh, G.M. Spinks, M.E. Kozlov, C.S. Haines, D. Suh, J. Foroughi, S.J. Kim, Y. Chen, T. Ware, M.K. Shin, L.D. Machado, A.F. Fonseca, J.D.W. Madden, W.E. Voit, D.S. Galvão, R.H. Baughman, Electrically, chemically, and photonically powered torsional and tensile actuation of hybrid carbon nanotube yarn muscles, Science (80-. ). 338 (2012) 928–932. https://doi.org/10.1126/science.1226762.

[26] M.O. Saed, C.P. Ambulo, H. Kim, R. De, V. Raval, K. Searles, D.A. Siddiqui, J.M.O. Cue, M.C. Stefan, M.R. Shankar, T.H. Ware, Molecularly‐Engineered 4D‐Printed Liquid Crystal Elastomer Actuators.pdf, Adv. Funct. Mater. 29 (2019). https://doi.org/10.1002/adfm.201806412.

[27] P. Chen, Y. Xu, S. He, X. Sun, S. Pan, J. Deng, D. Chen, H. Peng, Hierarchically arranged helical fibre actuators driven by solvents and vapours, Nat. Nanotechnol. 10 (2015) 1077–1083. https://doi.org/10.1038/nnano.2015.198.

[28] T. Jia, Y. Wang, Y. Dou, Y. Li, M.J. de Andrade, R. Wang, S. Fang, J. Li, Z. Yu, R. Qiao, Z. Liu, Y. Cheng, Y. Su, M. Minary-Jolandan, R.H. Baughman, D. Qian, Z. Liu, Moisture Sensitive Smart Yarns and Textiles from Self‐Balanced Silk Fiber Muscles.pdf, Adv. Funct. Mater. 29 (2019). https://doi.org/10.1002/adfm.201808241.

[29] J. Ayutsede, M. Gandhi, S. Sukigara, H. Ye, C. Hsu, Y. Gogotsi, F. Ko, Carbon Nanotube Reinforced Bombyx mori Silk Nanofibers by the Electrospinning Process, Biomacromolecules. 7 (2006) 208–214. https://doi.org/10.1021/bm0505888.

[30] H. Pan, Y. Zhang, Y. Hang, H. Shao, X. Hu, Y. Xu, C. Feng, Significantly Reinforced Composite Fibers Electrospun from Silk Fibroin/Carbon Nanotube Aqueous Solutions, Biomacromolecules. (2012). https://doi.org/10.1021/bm300877d.

[31] J. Wang, L. Li, M. Zhang, S. Liu, L. Jiang, Q. Shen, Directly obtaining high strength silk fiber from silkworm by feeding carbon nanotubes, Mater. Sci. Eng. C. 34 (2014) 417–421. https://doi.org/10.1016/j.msec.2013.09.041.

[32] G. Fang, Z. Zheng, J. Yao, M. Chen, Y. Tang, J. Zhong, Z. Qi, Z. Li, Z. Shao, X. Chen, Tough protein-carbon nanotube hybrid fibers comparable to natural spider silks, J. Mater. Chem. B,. 3 (2015) 3940–3947. https://doi.org/10.1039/c5tb00448a.

[33] Q. Wang, C. Wang, M. Zhang, M. Jian, Y. Zhang, Feeding Single-Walled Carbon Nanotubes or Graphene to Silkworms for Reinforced Silk Fibers, Nano Lett. 16 (2016) 6695–6700. https://doi.org/10.1021/acs.nanolett.6b03597.

[34] C. Zhang, Y. Zhang, H. Shao, X. Hu, Hybrid Silk Fibers Dry-Spun from Regenerated Silk Fibroin/Graphene Oxide Aqueous Solutions, ACS Appl. Mater. Interfaces. 8 (2016) 3349−3358. https://doi.org/10.1021/acsami.5b11245.
